# Sustained Control of Pyruvate Carboxylase by the Essential Second Messenger Cyclic di-AMP in Bacillus subtilis

**DOI:** 10.1128/mbio.03602-21

**Published:** 2022-02-08

**Authors:** Larissa Krüger, Christina Herzberg, Dennis Wicke, Patricia Scholz, Kerstin Schmitt, Asan Turdiev, Vincent T. Lee, Till Ischebeck, Jörg Stülke

**Affiliations:** a Department of General Microbiology, Institute for Microbiology & Genetics, GZMB, Georg-August-University Göttingen, Göttingen, Germany; b Department of Plant Biochemistry, GZMB, Georg-August-University Göttingen, Göttingen, Germany; c Department of Molecular Microbiology and Genetics, Service Unit LCMS Protein Analytics, Institute for Microbiology & Genetics, GZMB, Georg-August-University Göttingen, Göttingen, Germany; d Department of Cell Biology and Molecular Genetics, University of Maryland, College Park, Maryland, USA; University of Washington; Institut Pasteur

**Keywords:** *Bacillus subtilis*, cyclic di-AMP, TCA cycle, protein-protein interaction, pyruvate carboxylase, c-di-AMP, second messenger

## Abstract

In Bacillus subtilis and other Gram-positive bacteria, cyclic di-AMP is an essential second messenger that signals potassium availability by binding to a variety of proteins. In some bacteria, c-di-AMP also binds to the pyruvate carboxylase to inhibit its activity. We have discovered that in B. subtilis the c-di-AMP target protein DarB, rather than c-di-AMP itself, specifically binds to pyruvate carboxylase both *in vivo* and *in vitro*. This interaction stimulates the activity of the enzyme, as demonstrated by *in vitro* enzyme assays and *in vivo* metabolite determinations. Both the interaction and the activation of enzyme activity require apo-DarB and are inhibited by c-di-AMP. Under conditions of potassium starvation and corresponding low c-di-AMP levels, the demand for citric acid cycle intermediates is increased. Apo-DarB helps to replenish the cycle by activating both pyruvate carboxylase gene expression and enzymatic activity via triggering the stringent response as a result of its interaction with the (p)ppGpp synthetase Rel and by direct interaction with the enzyme, respectively.

## INTRODUCTION

In most organisms, the tricarboxylic acid (TCA) cycle is the central hub in metabolism. The cycle provides the cells with reducing power for respiration and to fuel anabolic reactions, and it generates precursors for important anabolic reactions, such as 2-oxoglutarate and oxaloacetate, for the biosynthesis of glutamate and aspartate and the derived amino acids, respectively. Accordingly, at least a partial TCA cycle is present in most organisms, with the notable exception of genome-reduced symbionts or pathogens that depend completely on their partner or host to acquire all metabolites.

The use of intermediates of the TCA cycles for anabolic purposes makes it essential to replenish the cycle to compensate for the loss of carbon backbones. The most important branch point of the cycle is 2-oxoglutarate, which can be either oxidized to succinyl-CoA within the TCA cycle or reduced to form glutamate, by far the most abundant metabolite in any living cell (1, 2). In particular, very high glutamate concentrations are required under conditions of osmotic stress when glutamate serves as the precursor for the production of molar levels of the osmoprotective amino acid proline (3). Moreover, the cells experience a high demand of glutamate under conditions of potassium limitation. Under this condition, positively charged amino acids derived from glutamate replace potassium to buffer the negative charge of the DNA (4). Accordingly, the cells are in high demand to replenish the carbon backbones for the TCA cycle under these conditions.

The Gram-positive model bacterium Bacillus subtilis has a complete TCA cycle. The TCA cycle in this bacterium is controlled at the levels of transcription and RNA degradation and by protein-protein interactions (5–9). The anaplerotic production of oxaloacetate from pyruvate to replenish the cycle is catalyzed by pyruvate carboxylase (PC), encoded by the *pycA* gene. Expression of the *pycA* gene is under positive stringent control, i.e., expression is increased under conditions of amino acid or potassium starvation as a result of the accumulation of the second messenger (p)ppGpp (10–12).

We are interested in signal transduction by the essential second messenger nucleotide cyclic di-AMP (c-di-AMP). In B. subtilis and many other bacteria, this nucleotide plays a key role in the control of potassium homeostasis and osmoadaptation by binding to transporters for potassium and osmoprotective compounds as well as to proteins and RNA molecules that regulate the expression of the corresponding genes (13–15). In addition to these factors involved in ion and osmotic homeostasis, c-di-AMP can bind to target proteins that do not have any direct enzymatic, transport, or regulatory functions. The receptor protein DarA has been identified in B. subtilis and the related bacteria Staphylococcus aureus and Listeria monocytogenes (16–18). This protein belongs to the PII superfamily of regulatory proteins (19); however, its function so far has remained elusive. The DarB receptor protein is present in B. subtilis and L. monocytogenes but not in S. aureus (17, 20). This protein consists of two CBS domains (named after cystathionine beta-synthase) that together form a so-called Bateman domain. These Bateman domains can bind a variety of adenosine nucleotides, including AMP, ADP, ATP, *S-*adenosyl methionine, NAD, and c-di-AMP (17, 20, 21). In many bacteria, c-di-AMP is essential for growth on complex media (13, 22). In B. subtilis, c-di-AMP is even essential on minimal media in the presence of potassium or glutamate (23, 24). While the toxicity of potassium or glutamate can easily be overcome by the acquisition of suppressor mutations, this is not possible on complex media. However, the deletion of either *darA* or *darB* in a B. subtilis strain lacking c-di-AMP (Δ*dac*) allows the emergence of suppressor mutations even on complex medium (24). This observation suggests that the DarA and DarB proteins are involved in harmful interactions in their c-di-AMP free apo-form. Indeed, the B. subtilis and L. monocytogenes DarB proteins can interact with the (p)ppGpp synthetase Rel in the absence of c-di-AMP and trigger the formation of (p)ppGpp and, thus, a stringent response (12, 25). c-di-AMP accumulates if the potassium levels in the cell are high, and the activation of Rel under conditions of potassium starvation links the availability of potassium, which is essential for ribosome function, to the stringent response and a global reprogramming of cellular activities.

Several studies have also implicated c-di-AMP signaling in the control of central metabolism. As mentioned above, c-di-AMP is essential if B. subtilis grows in the presence of glutamate, and the cellular c-di-AMP levels also respond to the presence of glutamate (24, 26). This glutamate toxicity at least partially results from activation of a low-affinity potassium transporter by glutamate and the very close interconnection between glutamate and potassium (2, 27). Moreover, in L. monocytogenes and in the lactic acid bacterium Lactococcus lactis, c-di-AMP binds to the pyruvate carboxylase and inhibits its enzymatic activity (17, 28, 29). By controlling the replenishment of the TCA cycle, c-di-AMP may play a major role in the regulation of cellular glutamate homeostasis.

In this work, we have serendipitously identified an interaction of the c-di-AMP target protein DarB with PC in B. subtilis. This interaction, which enhances the acetyl-coenzyme A (CoA)-dependent activation of PC activity, occurs with the apo-form of DarB, i.e., under conditions of potassium starvation if the cells experience a high demand for glutamate. While the molecular metabolism that links potassium availability and the intracellular c-di-AMP levels to PC activity are completely different in the closely related bacteria B. subtilis and L. monocytogenes, direct versus indirect with respect to c-di-AMP binding and positive or negative with respect to the effect on PC activity, they follow the same regulatory logic. As apo-DarB also triggers the stringent response by binding to Rel and, thus, the expression of the *pycA* gene encoding PC, c-di-AMP and DarB orchestrate the replenishment of the TCA cycle under conditions of potassium starvation at the levels of transcription and enzyme activity.

## RESULTS

### Identification of pyruvate carboxylase as an interaction partner of DarB.

**FIG 1:**
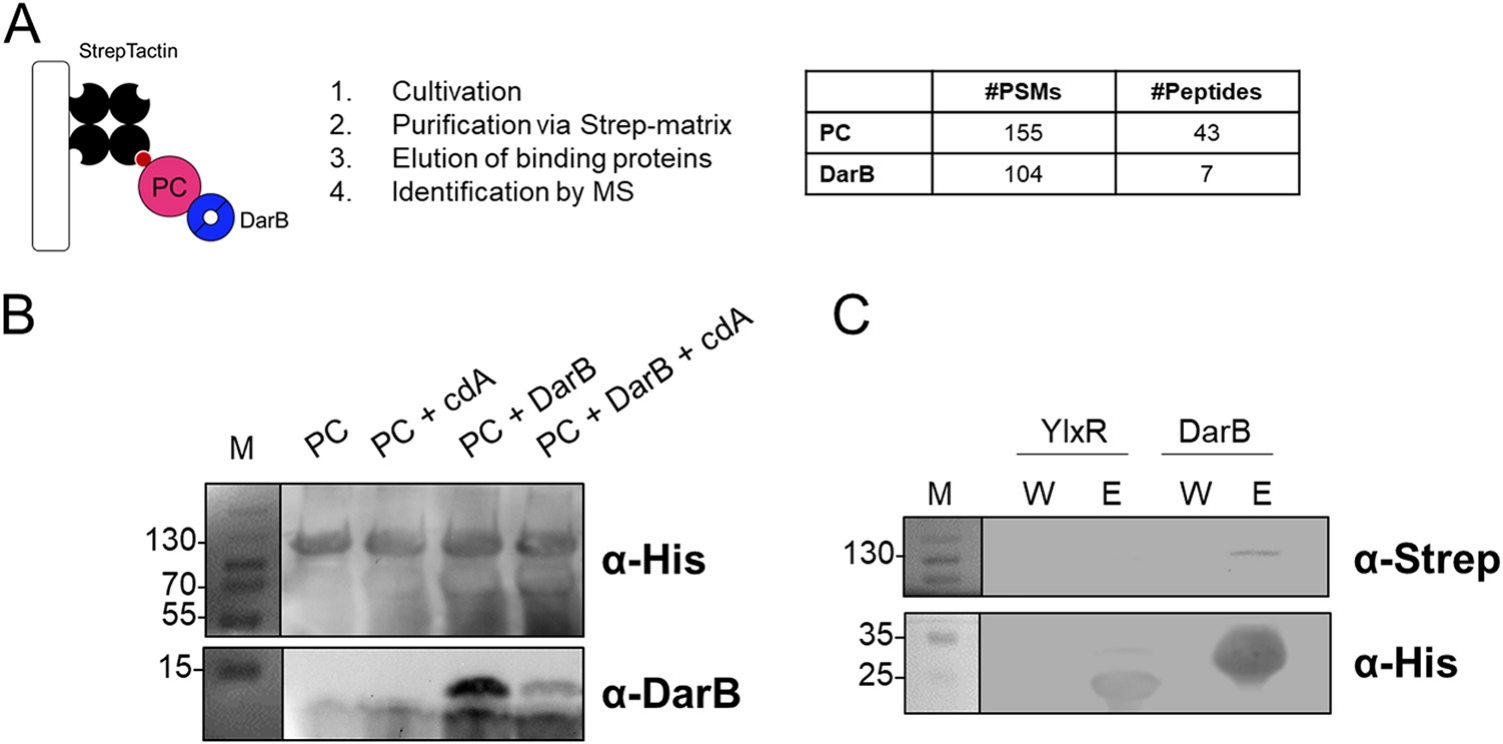
DarB interacts with PC. (A) Initial pulldown experiment. B. subtilis crude extract was purified via a StrepTactin matrix and analyzed by mass spectrometry, and the identified peptides are shown. (B) *In vitro* pulldown experiment with PC. Purified PC protein was immobilized onto a StrepTactin column and incubated with DarB, DarB preincubated with c-di-AMP, or the control protein BSA. The presence of DarB and PC in the elution fractions was analyzed by Western blotting with antibodies against DarB and the His tag of PC. BSA served as a negative control. The elution fractions were further analyzed by mass spectrometry. (C) Specificity of interaction between PC and DarB. His-tagged YlxR (negative control) and DarB were bound to a Ni-NTA column, and cell extract of B. subtilis expressing Strep-tagged PC was then passed over the columns. The binding of the bait proteins was verified by Western blotting using antibodies raised against the His tag, and coelution of Strep-PC was tested using antibodies raised against the Strep tag. Abbreviations: MS, mass spectrometry; PSMs, peptide sequence matches; cdA, c-di-AMP; M, marker; W, washing fraction; E, elution fraction.

In addition to the recently described interaction of DarB with the dual-function (p)ppGpp synthetase/hydrolase in B. subtilis, we serendipitously identified an interaction of DarB with pyruvate carboxylase (PC), a central metabolic enzyme that links glycolysis and the TCA cycle. This interaction was initially identified by passing a B. subtilis crude extract of cells grown in MSSM minimal medium over a StrepTactin column. PC and AccB, the biotin carboxyl carrier subunit of the acetyl-CoA carboxylase complex, contain biotin as a cofactor and, thus, intrinsically bind to the StrepTactin matrix. We analyzed the elution fractions for copurified proteins by mass spectrometry analysis (Fig. 1A). As expected, PC and AccB were identified with 155 and 9 peptide sequence matches (PSMs), respectively (Fig. 1A; see also Data Set S1A in the supplemental material). To our surprise, the DarB protein was the second highest-scoring protein, with 104 identified peptide sequence matches. In contrast to other coeluting proteins, DarB was not present in the washing fraction, indicating that its elution from the column was the result of an interaction with either PC or AccB. As the bait protein is usually identified with a higher number of PSMs than coeluting proteins, we assumed that DarB was bound to the column via PC rather than AccB, which was identified to a much lower extent.

10.1128/mbio.03602-21.6 DATA SET S1 Proteomic analysis of DarB-PC interaction. (A) Analysis of eluates from the initial pulldown experiment with Bacillus subtilis cell extract. (B) Analysis of eluates from the pulldown experiment with PC. (C) Analysis of eluates from the pulldown experiment with PC^CT+BCCP^. Download Data Set S1, PDF file, 0.2 MB. Copyright © 2022 Krüger et al. 2022 Krüger et al. https://creativecommons.org/licenses/by/4.0/ This content is distributed under the terms of the Creative Commons Attribution 4.0 International license.

To test the hypothesis that DarB interacts with pyruvate carboxylase, we assayed the binding of purified DarB to immobilized PC. For this, we purified the PC-6×His protein from E. coli and biotinylated it *in vitro* with purified BirA*^Bsu^* (Fig. S1A). Biotinylation of PC was verified via an avidin shift assay (Fig. S1B). For the pulldown, PC was coupled to a StrepTactin column, and DarB or YlxR, a control protein of similar size, was added. After extensive washing, the elution fractions were analyzed by Western blotting and mass spectrometry. Indeed, DarB was retained by and coeluted with PC (Fig. 1B and Fig. S2A and Data Set S1B). DarB was identified with 54 PSMs (Data Set S1B). In contrast, only 8 PSMs were found for the control protein YlxR. This result provides further evidence for the interaction between DarB and PC. To exclude the possibility of binding of DarB to the StrepTactin column, we passed biotinylated His-PC and DarB over the column and assayed the elution fractions for the presence of the proteins. As seen in prior experiments, His-PC was bound to the StrepTactin matrix whereas DarB was not (Fig. S2C), indicating that the elution of DarB depends on the presence of the interaction partner PC.

10.1128/mbio.03602-21.1 FIG S1 Purification of PC. (A) PC was purified in a two-step purification protocol first via an Ni-NTA-column and then purification of naturally biotinylated PC via a StrepTactin column. Unbiotinlyated PC was biotinylated *in vitro* by incubation with purified BirA protein. (B) The successful biotinylation of the previously unbiotinylated PC protein from the FT of the StrepTactin purification was tested with an avidin shift assay. Download FIG S1, PDF file, 0.5 MB. Copyright © 2022 Krüger et al. 2022 Krüger et al. https://creativecommons.org/licenses/by/4.0/ This content is distributed under the terms of the Creative Commons Attribution 4.0 International license.

10.1128/mbio.03602-21.2 FIG S2 DarB interacts with PC. SDS-PAGE from *in vitro* pulldown experiments with PC (A) and PC^CT+BCCP^ (B). Purified PC protein was immobilized onto a StrepTactin column and incubated with DarB, DarB preincubated with c-di-AMP, or the control protein YlxR. The presence of DarB and PC in the elution fractions was analyzed by SDS-PAGE, and the bands were excised from the gel (magenta boxes) and analyzed by mass spectrometry. The amount of DarB or YlxR identified in the excised gel bands is shown in the table. (C) Purified biotinylated His-PC and DarB were passed over a StrepTactin column, and the elution fractions were tested for the presence of His-PC (upper) and DarB (lower). Abbreviations: PSMs, peptide sequence matches; cdA, c-di-AMP. Download FIG S2, PDF file, 0.6 MB. Copyright © 2022 Krüger et al. 2022 Krüger et al. https://creativecommons.org/licenses/by/4.0/ This content is distributed under the terms of the Creative Commons Attribution 4.0 International license.

To get further evidence for the specific interaction between DarB and PC, we performed the interaction assay in an inverse fashion. For this purpose, His-tagged YlxR (as a negative-control protein) and DarB were bound to a nickel-nitrilotriacetic acid (Ni-NTA) column, and a cell extract of B. subtilis carrying the expression plasmid pGP1289 for constitutive expression of Strep-tagged PC was passed over the columns. The presence of the bait proteins and PC in the elution fractions was assayed by Western blotting using antibodies raised against the His or Strep tag, respectively. As shown in Fig. 1C, PC did not coelute with the control protein YlxR, whereas coelution was observed when DarB was bound to the matrix. Thus, DarB and PC specifically interact with each other.

DarB is a c-di-AMP receptor protein, and these proteins are considered to function in response to the presence of the ligand, and indeed we have shown previously that c-di-AMP prevents interaction of DarB with Rel (12). To study the importance of the second messenger c-di-AMP for the interaction of DarB with PC, we also tested the ability of DarB to bind PC in the presence of c-di-AMP in the pulldown assay described above. As observed for Rel, DarB bound PC to a much lesser extent (18 PSMs) in the presence of the second messenger, indicating that the presence of c-di-AMP reduces the interaction (Fig. 1B and Fig. S2A, Data set S1B).

Taken together, our results demonstrate a specific interaction between PC and the apo form of DarB. In contrast, the presence of c-di-AMP interferes with the interaction between the two proteins.

### DarB interacts with the C-terminal part of PC.

**FIG 2:**
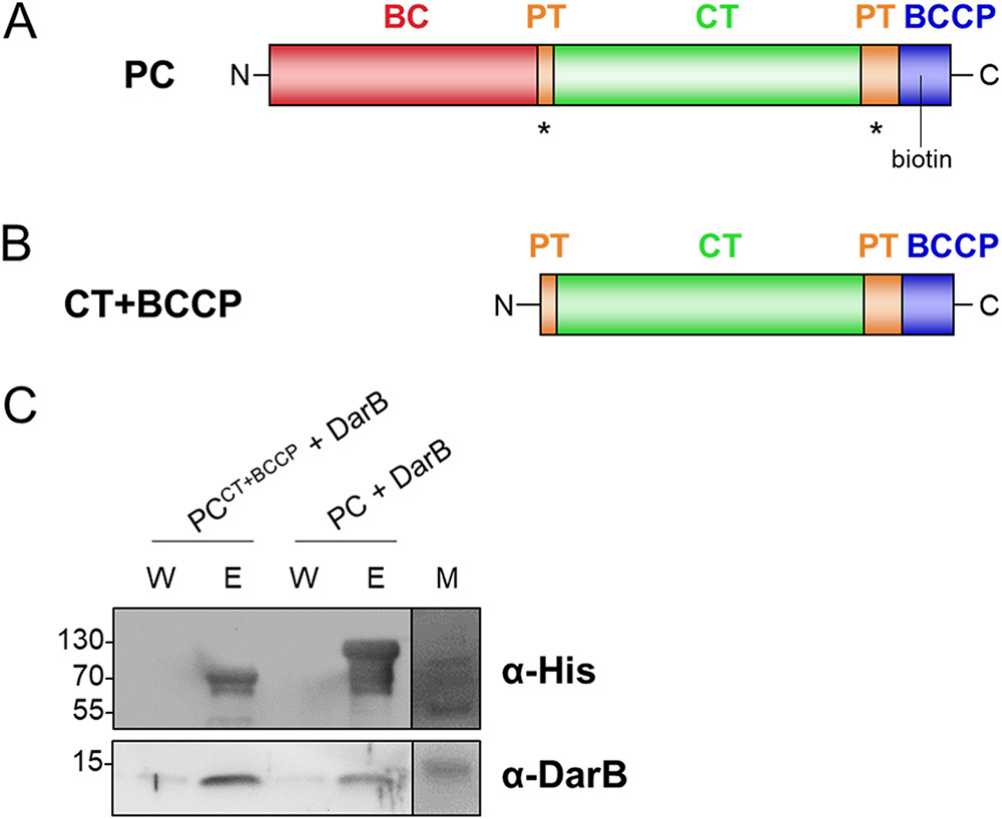
DarB interacts with the C-terminal part of PC. (A) Domain organization of pyruvate carboxylase. An asterisk indicates the binding sites for acetyl-CoA (33). (B) Domain organization of the CT+BCCP mutant. (B) *In vitro* pulldown experiment with PC^CT+BCCP^. Purified PC^CT+BCCP^ protein was immobilized onto a StrepTactin column and incubated with DarB or the control protein, BSA. The presence of DarB and PC in the elution fractions was analyzed by Western blotting with antibodies against DarB and the His tag of PC. BSA served as a negative control. The elution fractions were further analyzed by mass spectrometry. Abbreviations: BC, biotin carboxylation domain; PT, PC tetramerization domain; CT, carboxyltransferase domain; BCCP, biotin carboxyl carrier protein; E, elution fraction; W, wash fraction.

Pyruvate carboxylase is a multifunctional tetrameric protein composed of three functional domains, the N-terminal biotin carboxylase (BC), the central carboxyl transferase (CT), and the C-terminal biotin carboxyl carrier protein (BCCP) domains (Fig. 2A). To identify the PC domain that is responsible for the interaction with DarB, we generated and purified a truncated PC protein in which we deleted the BC domain (Fig. 2B). The ability of this truncated protein consisting of the CT and BCCP domains to interact with DarB was then tested again in the pulldown assay. Deletion of the BC domain did not prevent the interaction, as DarB still coelutes with the truncated PC (Fig. 2C and Fig. S2B and Data Set S1C). This indicates that DarB binds to the C-terminal part of PC, whereas the BC domain is not involved in the interaction with DarB. Interestingly, PC*^Lmo^* binds c-di-AMP at the CT domain to inhibit the enzymatic activity (17).

### PC does not bind c-di-AMP.

In some bacteria, including L. monocytogenes and L. lactis, the pyruvate carboxylase is subject to regulation by c-di-AMP (17, 28). Thus, we wondered whether the B. subtilis enzyme would also directly interact with the second messenger. To study this, we expressed PC and the c-di-AMP target KdpD*^Lmo^* as a positive control in E. coli, and the lysates of strains carrying the corresponding plasmids were assessed for a possible interaction with c-di-AMP using the differential radial capillary action of ligand assay (DRaCALA). While c-di-AMP clearly bound to the known target protein KdpD (30), no binding of the second messenger to PC was observed (Fig. S3).

10.1128/mbio.03602-21.3 FIG S3 E. coli whole-cell lysates overexpressing KdpD but not PycA bind c-di-AMP. (A) Fraction bound of radiolabeled ^32^P-c-di-AMP from DRaCALA experiments is shown for lysates from E. coli induced for the expression of the indicated genes in the presence of 100 μM nonspecific ATP competitor. Three independent lysates were prepared and analyzed for each indicated gene and vector controls. (B) Protein expression in E. coli lysates induced for expression of the indicated genes used in DRaCALA. Proteins were analyzed by SDS-PAGE and stained by Coomassie brilliant blue. Download FIG S3, PDF file, 0.6 MB. Copyright © 2022 Krüger et al. 2022 Krüger et al. https://creativecommons.org/licenses/by/4.0/ This content is distributed under the terms of the Creative Commons Attribution 4.0 International license.

Our results demonstrate that c-di-AMP does not directly bind to PC, unlike PC from other bacteria, such as L. monocytogenes and L. lactis (17, 28). Instead, the second messenger affects the protein-protein interaction between DarB and PC. This suggests that c-di-AMP-mediated control of pyruvate carboxylase activity is achieved in a distinct way in B. subtilis.

### DarB is an activator of PC.

Based on the results presented above, we hypothesized that in B. subtilis, PC activity might be regulated by the c-di-AMP receptor DarB rather than directly by c-di-AMP, as described for L. monocytogenes and L. lactis (17, 28). To test this idea, we used purified PC to assay the formation of oxaloacetate.

**FIG 3:**
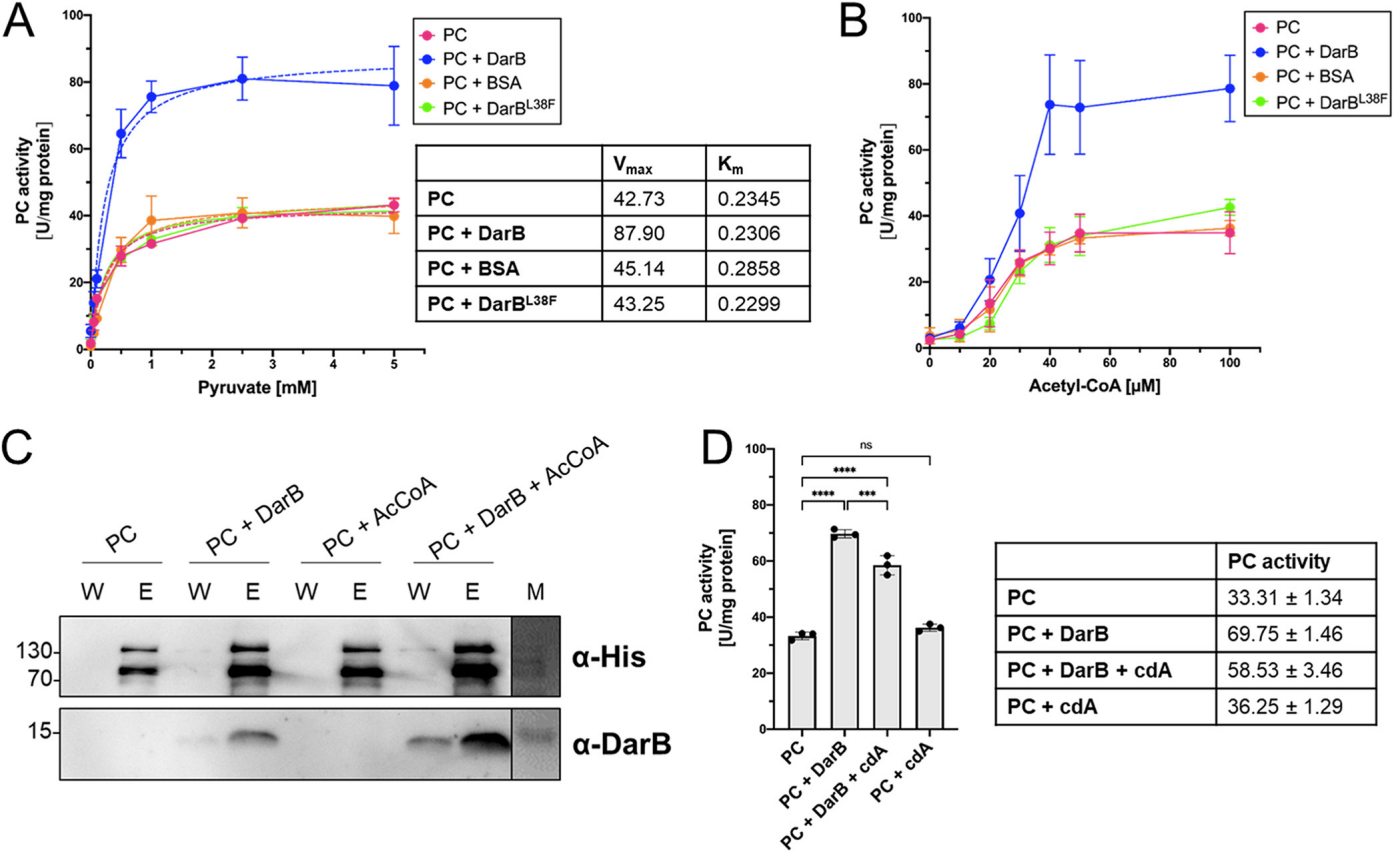
DarB stimulates the synthesis of oxaloacetate. The activity of PC was assessed in an *in vitro* activity assay. (A) The reaction mix contained 0.26 μM PC, 2.6 μM DarB/BSA/DarB^L38F^ (measured based on monomer), 100 μM acetyl-CoA, 2 mM ATP, 25 mM KHCO_3_, and the indicated pyruvate concentrations. The catalytic activity of PC toward pyruvate obeys Michaelis–Menten kinetics. (B) PC is activated by acetyl-CoA and DarB, and activation by DarB is acetyl-CoA dependent. The reaction mix contained 0.26 μM PC and 2.6 μM DarB/BSA/DarB^L38F^, 25 mM KHCO_3_, 2.5 mM pyruvate, and various acetyl-CoA concentrations. (C) c-di-AMP inhibits activation by DarB to 16%. cdA itself has no influence on PC. The reaction mix contained 0.26 μM PC, 2.6 μM DarB, 25 mM KHCO_3_, 2.5 mM pyruvate, 100 μM acetyl-CoA, and 26 μM cdA if indicated. The experiment was conducted with *n* = 3 biologically independent samples. (D) c-di-AMP inhibits activation by DarB to 16%. cdA itself has no influence on PC. The reaction mix contained 0.26 μM PC, 2.6 μM DarB, 25 mM KHCO_3_, 2.5 mM pyruvate, 100 μM acetyl-CoA, and 26 μM cdA if indicated. Data are presented as mean values ± standard deviations. Statistical analysis was performed using a one-way analysis of variance, followed by Tukey’s multiple-comparison test (****, *P < *0.0001). cdA, c-di-AMP.

The purified PC behaved similarly to published data in a malate dehydrogenase-coupled standard spectrophotometric assay in the presence of acetyl-CoA as the known effector metabolite (31). We determined a *K_m_* for pyruvate of 0.23 mM (Fig. 3A). Interestingly, the substrate affinity was not altered in the presence of DarB (0.23 mM) (Fig. 3A). We measured a *V*_max_ of 42.7 and 87.9 U·mg protein^−1^ for PC in the absence and presence of DarB, respectively (Fig. 3A). Typically, allosteric enzyme activators affect only one of the two Michaelis-Menten parameters and either lower the *K_m_* or raise the *V*_max_. Thus, DarB seems to act as an activator of PC, as its presence increases the *V*_max_ 2-fold. The presence of the control protein bovine serum albumin (BSA) does not affect the enzymatic activity of PC, indicating that the increase in the *V*_max_ in the presence of DarB is specific.

The carboxylation of pyruvate serves to generate one of the two substrates for citrate synthesis. The second substrate, acetyl-CoA, is an allosteric activator of PC (32, 33). PC proteins from different bacteria show different levels of acetyl-CoA dependence. While the catalytic activity of L. lactis PC is not affected by acetyl-CoA (28), it plays a significant role in the activation of the enzyme in B. subtilis, L. monocytogenes, and Rhodobacter capsulatus (17, 34, 35). We asked whether acetyl-CoA and DarB exert their effects on PC independently or whether they act in a coordinated manner. To address this question, we determined PC activity in the presence of various acetyl-CoA concentrations (Fig. 3B). As described previously (34), the activity of PC was strongly enhanced in the presence of acetyl-CoA. In the absence of acetyl-CoA, DarB did not affect PC activity, whereas the acetyl-CoA-dependent activation of the enzymatic activity was further enhanced if DarB was present. In contrast, BSA, which served as a control protein, did not affect PC activity irrespective of the availability of acetyl-CoA. Thus, the data indicate that DarB functions as a coactivator of PC activity. To get a deeper insight into this observation, we repeated the pulldown experiments in the presence of acetyl-CoA. Indeed, the presence of acetyl-CoA increased the amount of DarB retained on the column by immobilized PC (Fig. 3C). This enhanced interaction between the two proteins in the presence of acetyl-CoA may explain the cumulative activation of PC activity by both acetyl-CoA and DarB.

The data presented above show that c-di-AMP interferes with the interaction of DarB with PC (Fig. 1B). We therefore also tested the effect of the second messenger on the activation of PC by DarB. As shown in Fig. 3D, the presence of c-di-AMP reduces the activation of PC by DarB. In agreement with the results from the DRaCALA experiments showing that PC is unable to bind c-di-AMP (Fig. S3), the presence of the second messenger alone did not affect PC activity (Fig. 3D), demonstrating that the decrease in activation is mediated by c-di-AMP binding to DarB.

### DarB is unable to overcome the inhibition of PC by aspartate.

In addition to the activation by acetyl-CoA, PC is subject to allosteric inhibition by aspartate (36). Aspartate functions as a regulatory feedback inhibitor that responds to increased levels of TCA cycle intermediates. As aspartate was previously shown to inhibit the enzyme competitively with respect to acetyl-CoA (36), we asked whether aspartate might also interfere with the activation by DarB. PC assays demonstrated that DarB was no longer able to activate PC in the presence of aspartate (Fig. S4).

10.1128/mbio.03602-21.4 FIG S4 Biochemical characterization of PC regulation by aspartate. The reaction mix contained 0.26 μM PC, 2.6 μM DarB or BSA (based on monomer), 10 mM Tris (pH 7.8), 150 mM KCl, 5 mM MgCl_2_, 10 U malate dehydrogenase, 0.4 mM NADH, 25 mM KHCO_3_, 2.5 mM pyruvate, 100 μM acetyl-CoA, and 16 mM aspartate if indicated. The graph shows the consumption of NADH over time, which is proportional to the synthesis of oxaloacetate by PC. Download FIG S4, PDF file, 0.5 MB. Copyright © 2022 Krüger et al. 2022 Krüger et al. https://creativecommons.org/licenses/by/4.0/ This content is distributed under the terms of the Creative Commons Attribution 4.0 International license.

### Differential effects of a mutation in the interaction surface of DarB on the interaction with PC.

To further validate the regulatory interaction of DarB with PC, we mutated the DarB protein at its interaction surface (12, 25) (PDB entry 6YJA). Specifically, we changed Leu38 to Phe. DarB^L38F^ was tested for c-di-AMP binding interaction with the known DarB target proteins, Rel and PC, for its effect on PC activity and for binding of acetyl-CoA. Isothermal titration calorimetry (ITC) revealed that the mutant protein binds c-di-AMP with a similar affinity as the wild-type protein (Fig. S5A), indicating that it folds correctly. To verify that the L38F mutation is located at the interaction surface, we tested the ability of the DarB mutant to bind to its initially identified interaction partner, the Rel protein. We observed that the L38F mutation abolished the ability to bind Rel, whereas the wild-type DarB protein efficiently bound Rel (Fig. S5B). Thus, the mutant protein folds correctly but has lost the ability to interact with the Rel protein, confirming that the mutation affects the interaction surface of DarB. To investigate whether the interaction with PC is also affected by the mutation, we tested the ability of the mutant protein to interact with PC in the *in vitro* pulldown experiment. Surprisingly, the mutant protein was still able to bind to PC in the pulldown experiment (Fig. S5C), suggesting that the single L38F amino acid exchange is not sufficient to abolish the interaction. However, when we tested the DarB^L38F^ mutant protein in the activity assay, we observed that it is unable to activate PC (Fig. 3A and B). This counterintuitive result suggests that highly specific interactions between particular residues of the two proteins are required for the activation of PC.

10.1128/mbio.03602-21.5 FIG S5 Analysis of the binding affinities of the DarB mutant L38F towards c-di-AMP and Rel^NTD^. (A) The ability of the DarB^L38F^ mutant protein to bind c-di-AMP was assessed by isothermal titration calorimetry (ITC). The cell and the syringe contained 10 μM DarB^L38F^ and 100 μM c-di-AMP, respectively. Titration profiles and the determined molar ratio are shown together with the calculated *K_D_* value for binding of c-di-AMP as well as the determined number of ligand binding sites. For comparison, the calculated *K_D_* value and number of ligand binding sites from wild-type DarB from Krüger et al. (27) are shown in the table as well. (B) The ability of the DarB^L38F^ mutant (green) to bind Rel was assessed by ITC. The cell and the syringe contained 10 μM Rel^NTD^ and 100 μM DarB^L38F^, respectively. For comparison, the titration profile of wild-type DarB into Rel was taken from Krüger et al. (27) (blue). Titration profiles and the determined molar ratios are shown. (C) Silver stain of SDS-PAGE from *in vitro* pulldown experiments with PC. Purified PC protein was immobilized onto a StrepTactin column and incubated with DarB or the DarB^L38F^ mutant. The elution fractions were analyzed by SDS-PAGE. The magenta box shows the band corresponding to the DarB protein. cdA, c-di-AMP. Download FIG S5, PDF file, 0.6 MB. Copyright © 2022 Krüger et al. 2022 Krüger et al. https://creativecommons.org/licenses/by/4.0/ This content is distributed under the terms of the Creative Commons Attribution 4.0 International license.

### Overexpression of DarB results in *in vivo* changes in metabolite concentration.

**FIG 4:**
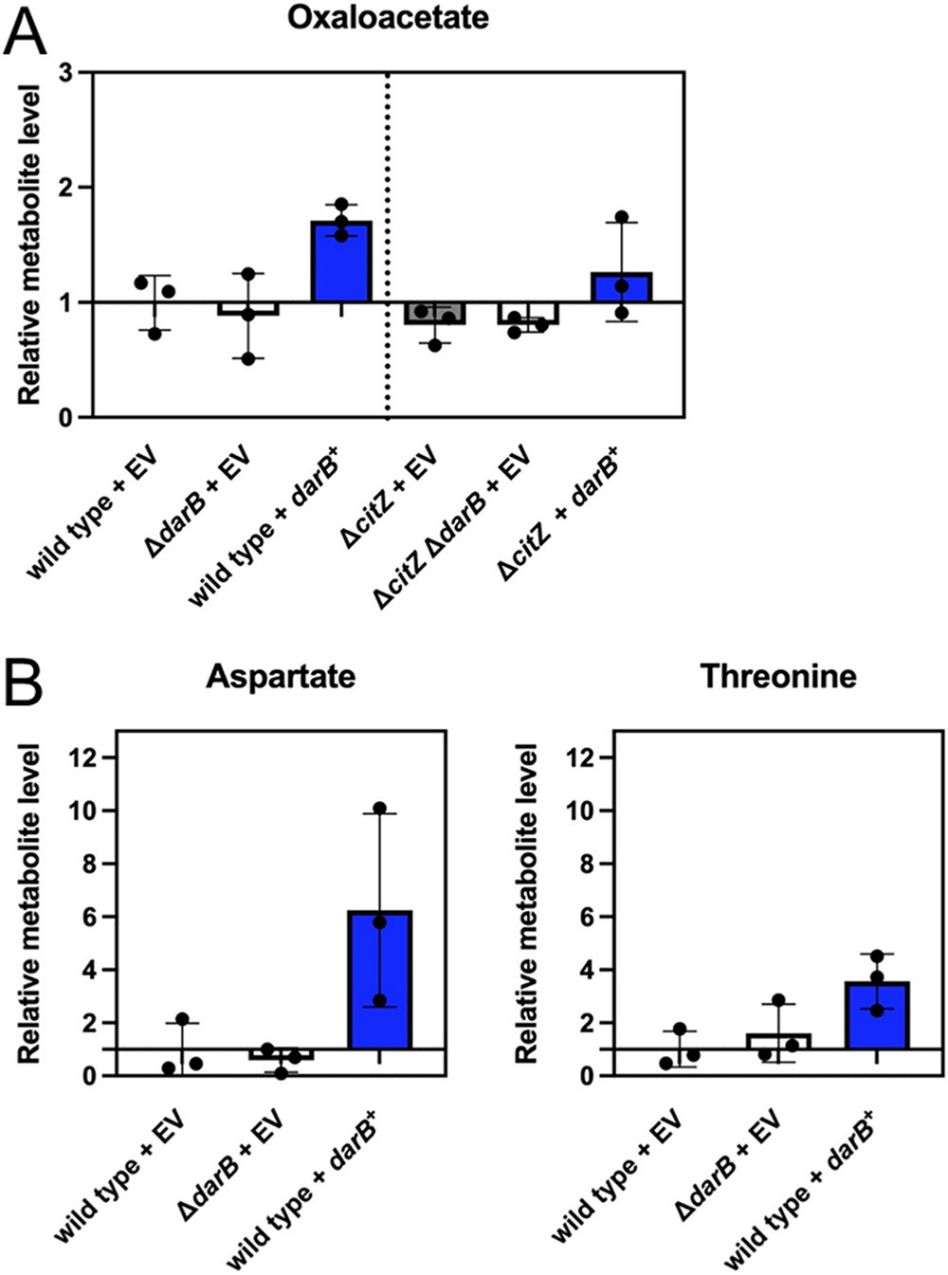
DarB affects the synthesis of amino acids derived from oxaloacetate. Determination of intracellular metabolite levels in the wild type, a *darB* mutant, and a strain overexpressing *darB* by GC-MS. The metabolites were quantified relative to the wild type. The experiments were performed with three biological replicates. (A) Determination of oxaloacetate in derivatives of the wild-type strain 168 and of the *citZ* mutant lacking citrate synthase. (B) Determination of intracellular levels of the oxaloacetate-derived amino acids aspartate and threonine. EV, empty vector control.

The data presented above suggest that the interaction of DarB with PC results in an increase of the intracellular oxaloacetate levels. To verify this assumption, we compared the intracellular oxaloacetate concentrations in a wild-type strain, the *darB* mutant, and the *darB*^+^ strain overexpressing DarB. Due to the high activity of citrate synthase, freshly synthesized oxaloacetate is quickly metabolized to citrate. To allow accumulation of oxaloacetate, we performed these measurements in a *citZ* mutant strain that lacks citrate synthase and is therefore unable to use oxaloacetate for citrate synthesis. However, the levels of oxaloacetate were still close to the detection limit (Fig. 4A). A previous study suggested that the effect of increased expression of *pycA* could only be detected by measuring the accumulation of the oxaloacetate-derived amino acids aspartate and threonine (10). Indeed, the levels of these amino acids were increased in the *darB* overexpression strain (Fig. 4B). In contrast, the deletion of *darB* set their relative levels back to the wild-type situation. These findings support the conclusion that DarB activates PC activity.

## DISCUSSION

The data presented in this study identify the c-di-AMP receptor protein DarB as a bona fide activator of PC. The interaction between the two proteins enhances enzyme velocity in the presence of the known allosteric activator acetyl-CoA. This additional activation of PC ensures that the TCA cycle can operate even under conditions of high demand for specific intermediates. Under most circumstances, the withdrawal of intermediates for anabolic purposes is rather constant; however, under conditions of potassium starvation a substantial part of the 2-oxoglutatarate formed in the TCA cycle is needed to produce glutamate as the precursor for positively charged amino acids that replace potassium in buffering the negative charge of DNA (4). Thus, it is important to couple the replenishment of the TCA cycle to potassium availability.

The control of the anaplerotic reaction catalyzed by PC is achieved by the c-di-AMP receptor protein DarB in B. subtilis. Under conditions of potassium starvation, the c-di-AMP level is low, and apo-DarB binds and activates PC, allowing replenishment of TCA cycle intermediates under conditions of high demand. Interestingly, c-di-AMP also controls PC activity in L. monocytogenes and L. lactis. However, unlike what was observed here for B. subtilis, in those bacteria, c-di-AMP, which accumulates if the potassium supply is sufficient (23), directly binds to PC and inactivates the enzyme (17, 28). Even though the exact mechanism of regulation differs between species, the outcome, higher PC activity in the absence of potassium, is the same.

The presence of c-di-AMP interferes with the interaction between DarB and PC and, thus, with PC activation. It is interesting that the recently determined structure of DarB in the presence of c-di-AMP (PDB entry 6YJA) suggests that one adenine of the bound c-di-AMP is buried in the DarB dimer interface, whereas the second adenine protrudes out of the protein. This immediately suggests that this protruding adenine prevents DarB binding to PC in a sting-like manner.

B. subtilis strains lacking c-di-AMP are unable to grow on complex medium. Such strains can acquire suppressor mutations that allow growth on complex medium only in the absence of either of the c-di-AMP-binding signal transduction proteins, DarA or DarB (24). This observation suggests that apo-DarA and apo-DarB are engaged in growth inhibiting interactions under these conditions. Indeed, the activation of PC by apo-DarB might fuel a futile cycle and result in the loss of energy as the reaction requires the hydrolysis of ATP. In complex medium, cells have all amino acids in excess, so the DarB-mediated wasteful activation of PC as well as of the Rel protein is indeed likely to result in growth inhibition.

**FIG 5:**
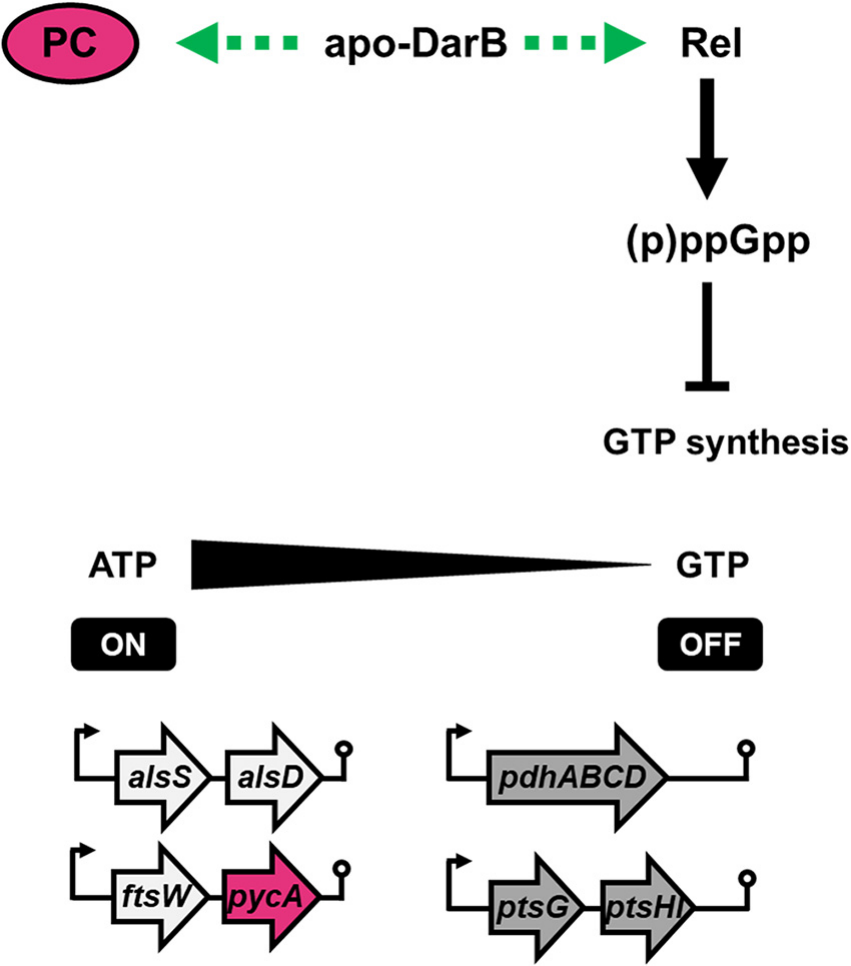
Control of PC activity by DarB follows the model of sustained sensing. DarB regulates the activity of PC at two stages: (i) by direct binding to the enzyme and activation and (ii) indirectly by activation of the stringent response and increased expression of the *pycA* transcript resulting from the interaction with the (p)ppGpp synthetase/hydrolase Rel.

The results reported here and in previous studies establish that DarB modulates the activities of at least two enzymes under conditions of potassium limitation: DarB activates Rel (12, 25), resulting in the accumulation of (p)ppGpp and an induction of the stringent response. A part of this response is the increased expression of the *pycA* gene encoding PC. Thus, DarB regulates pyruvate carboxylation at two levels, i.e., (i) directly at the protein level by binding to the enzyme and stimulating the synthesis of oxaloacetate and (ii) indirectly by interaction with Rel, which leads to activation of the stringent response and to the increased expression of the *pycA* gene (Fig. 5). This double activation of PC faithfully ensures the production of oxaloacetate and, thus, the continued operation of the TCA cycle, the core of B. subtilis metabolism, under conditions of potassium starvation. The control of one biological process by a signaling molecule that binds different targets to regulate multiple steps of the process has been termed sustained sensing (37). In this case, c-di-AMP controls DarB in a negative manner for both its activities, and the regulatory interactions between DarB and Rel or PC are inhibited by c-di-AMP during growth under potassium-sufficient conditions. This may also explain why these regulatory processes have not been discovered earlier. The regulation of pyruvate carboxylase is the second example of c-di-AMP-mediated sustained sensing in B. subtilis in addition to the control of potassium homeostasis by direct binding of c-di-AMP to potassium transporters and riboswitches that control their expression (20, 23) (Fig. 5). It will be interesting to test whether other c-di-AMP-controlled processes, such as the uptake of osmoprotective compounds (14, 20, 38, 39), are also subject to dual control of gene expression and protein activity.

A striking feature of c-di-AMP signaling via dedicated signal transduction proteins is that the interactions take place between the apo-proteins and their partners. For both DarA and DarB, a growth-inhibitory effect on complex medium has been observed in B. subtilis and L. monocytogenes if the cells are unable to produce c-di-AMP (24, 40), indicating that the apo-proteins are involved in interactions. Moreover, two interaction partners have so far been discovered for DarB: this protein binds to the Rel protein in both B. subtilis and L. monocytogenes (12, 25), and, as described in this study, B. subtilis DarB also interacts with PC. In both cases, binding of apo-DarB, which is present under conditions of potassium starvation, controls the activities of the partner proteins. In principle, the control of the DarB partner in response to potassium starvation could also occur by binding of c-di-AMP, resulting in the inactivation of the target protein. Indeed, this was observed for PC of L. monocytogenes and L. lactis (17, 28). Alternatively, the c-di-AMP-bound receptor proteins might interact with their targets to inactivate them. Of the three scenarios, interaction of the apo c-di-AMP receptors with their targets might be preferred, since the evolution of protein interaction surfaces may have fewer constraints than the evolution of second-messenger binding pockets that require binding under standard conditions (potassium sufficiency). Strikingly, in cyanobacteria, the c-di-AMP receptor protein SbtB binds and activates its target, GlgB, an enzyme required for glycogen synthesis in the c-di-AMP-bound state (41). It will be interesting to see whether future research will discover DarB targets that interact with the c-di-AMP-bound form of DarB and whether, for the other c-di-AMP binding signal transduction proteins, the interactions also occur with the apo-proteins.

## MATERIALS AND METHODS

### Strains, media, and growth conditions.

E. coli DH5α and Rosetta(DE3) (42) were used for cloning and for the expression of recombinant proteins, respectively. All B. subtilis strains used in this study are derivatives of the laboratory strain 168. B. subtilis and E. coli were grown in Luria-Bertani (LB) or in sporulation (SP) medium (42, 43). For growth assays and the *in vivo* interaction experiments, B. subtilis was cultivated in LB or MSSM (23). MSSM is a modified SM medium in which KH_2_PO_4_ was replaced by NaH_2_PO_4_ and KCl was added as indicated. The media were supplemented with ampicillin (100 μg/ml), kanamycin (50 μg/ml), chloramphenicol (5 μg/ml), or erythromycin and lincomycin (2 and 25 μg/ml, respectively) if required.

### DNA manipulation.

Transformation of E. coli and plasmid DNA extraction were performed using standard procedures (42). All commercially available plasmids, restriction enzymes, T4 DNA ligase, and DNA polymerases were used as recommended by the manufacturers. B. subtilis was transformed with plasmids, genomic DNA, or PCR products according to the two-step protocol (43). Transformants were selected on LB plates containing erythromycin (2 μg/ml) plus lincomycin (25 μg/ml), chloramphenicol (5 μg/ml), kanamycin (10 μg/ml), or spectinomycin (250 μg/ml). DNA fragments were purified using the QIAquick PCR purification kit (Qiagen, Hilden, Germany). DNA sequences were determined by the dideoxy chain termination method (42). Introduction of mutations in the *darB* allele was achieved by the combined chain reaction by using an additional 5′ phosphorylated primer to introduce the mutation (44).

### Construction of mutant strains by allelic replacement.

Deletion of the *citZ* gene was achieved by transformation of B. subtilis 168 with a PCR product constructed using oligonucleotides to amplify DNA fragments flanking the target genes and an appropriate intervening resistance cassette as described previously (45). The integrity of the regions flanking the integrated resistance cassette was verified by sequencing PCR products of about 1,100 bp amplified from chromosomal DNA of the resulting mutant strains, GP797. All strains and oligonucleotides are listed in Tables S1 and S3, respectively, in the supplemental material.

10.1128/mbio.03602-21.7 TABLE S1 Strains used in this study. Download Table S1, PDF file, 0.07 MB. Copyright © 2022 Krüger et al. 2022 Krüger et al. https://creativecommons.org/licenses/by/4.0/ This content is distributed under the terms of the Creative Commons Attribution 4.0 International license.

10.1128/mbio.03602-21.9 TABLE S3 Oligonucleotides used in this study. Download Table S3, PDF file, 0.08 MB. Copyright © 2022 Krüger et al. 2022 Krüger et al. https://creativecommons.org/licenses/by/4.0/ This content is distributed under the terms of the Creative Commons Attribution 4.0 International license.

### Plasmid constructions.

The *pycA*, *darB-L38F*, and *ylxR* alleles were amplified using chromosomal DNA of B. subtilis 168 as the template and appropriate oligonucleotides that attached specific restriction sites to the fragment. Those were XbaI and XhoI for cloning *pycA* in pET28a (Novagen, Germany) and BsaI and XhoI for cloning *darB-L38F* and *ylxR* in pET-SUMO (Invitrogen, Germany). The truncated *pycA* variants were constructed as follows. *pycA*^CT+BCCP^ contained amino acids 461 to 1148 and was cloned into pET28a by adding XbaI and XhoI restriction sites. For purification of Strep-PC from B. subtilis extracts, the *pycA* gene was cloned between the XbaI and SphI restriction sites of pGP380 (46). The B. subtilis *birA* gene was amplified and the PCR product was cloned between the BamHI and SalI sites of the expression vector pWH844 (47). All plasmids are listed in Table S2.

10.1128/mbio.03602-21.8 TABLE S2 Plasmids used in this study. Download Table S2, PDF file, 0.08 MB. Copyright © 2022 Krüger et al. 2022 Krüger et al. https://creativecommons.org/licenses/by/4.0/ This content is distributed under the terms of the Creative Commons Attribution 4.0 International license.

### Protein expression and purification.

E. coli Rosetta(DE3) was transformed with the plasmid pGP2972, pGP3481, pGP3618, pGP3458, pGP3455, or pGP2690, encoding wild-type or mutant 6×His-SUMO-DarB for purification of DarB, DarB^L38F^, YlxR, wild-type and truncated PC-6×His and PC^CT+BCCP^-6×His, or BirA. For overexpression of DarB, DarB^L38F^, YlxR, and BirA, cells were grown in 2× LB, and expression was induced by the addition of isopropyl 1-thio-β-d-galactopyranoside (IPTG; final concentration, 1 mM) to exponentially growing cultures (optical density at 600 nm [OD_600_] of 0.8). The His-tagged proteins DarB, DarB^L38F^, YlxR, and BirA were purified in 1× ZAP buffer (50 mM Tris-HCl, 200 mM NaCl, pH 7.5 [pH 8.3 for 6×His-BirA]). Cells were lysed by four passes (18,000 lb/in^2^) through an HTU DIGI-F press (G. Heinemann, Germany). After lysis, the crude extract was centrifuged at 46,400 × *g* for 60 min and then passed over a Ni^2+^ nitrilotriacetic acid column (IBA, Göttingen, Germany). The proteins were eluted with an imidazole gradient. After elution, the fractions were tested for the desired protein using SDS-PAGE. For the purification of BirA, the column was washed with two column volumes of 4 M LiCl to remove DNA prior to elution of the protein with 20 mM and 100 mM imidazole. BirA was dialyzed against 1× ZAP buffer (pH 8.3, 5% glycerol). To remove the SUMO tag from the 6×His-SUMO-tagged proteins, the relevant fractions were combined, and the SUMO tag was removed with the SUMO protease (ratio, 100:1) during overnight dialysis against 1× ZAP buffer (pH 7.5). The cleaved SUMO moiety and the protease were removed using a Ni^2+^ nitrilotriacetic acid column (IBA), and the proteins were dialyzed against 1× ZAP buffer (pH 7.5).

PC was overexpressed in E. coli Rosetta(DE3) cultures carrying the plasmids encoding wild-type and the truncated PC in 2× LB at 37°C. When the cultures reached an OD_600_ of 1.2, MnCl_2_ was added (final concentration, 10 mM) and the cultures were transferred to 28°C. After 20 min, expression of the recombinant proteins was induced by the addition of isopropyl 1-thio-β-d-galactopyranoside (final concentration, 0.3 mM), and the cultures were grown for 3 h. PC-6×His and PC^CT+BCCP^-6×His were purified as described previously (48) in buffer A (20 mM Tris, pH 7.8, 200 mM NaCl, 0.5 mM EGTA, 6 mM β-mercaptoethanol). The cells were lysed and purified as described above. PC and PC^CT+BCCP^ were dialyzed against dialysis buffer (10 mM Tris, pH 7.8, 150 mM KCl, 6 mM β-mercaptoethanol, 5% glycerol). The purified proteins were concentrated in a Vivaspin turbo 15 (Sartorius) centrifugal filter device (cutoff of 5 or 50 kDa). The protein samples were stored at −80°C until further use. The protein concentration was determined according to the method of Bradford (49) using the Bio-Rad dye binding assay and bovine serum albumin as the standard.

### Biotinylation of PC and PC^CT+BCCP^.

Biotinylation of PC and PC^CT+BCCP^ was achieved by incubation of PC (25 μM) with purified BirA (50 μM) in 1× ZAP buffer (pH 8.3) containing 2 mM ATP, 5 mM MgCl_2_, and 0.15 mM biotin. The reaction mixture was incubated overnight in the fridge, and biotinylation of the protein was tested with an avidin shift assay. For this purpose, 4 μl of the reaction mixture together with SDS-loading dye were boiled for 5 min at 95°C. Afterwards, 36 μl avidin (1.2 mg/ml) was added and the sample was incubated at 25°C for 40 min. A sample with buffer instead of avidin served as a control. Binding of avidin to the biotinylated PC was visualized via an SDS-PAGE. The interaction between avidin and biotin is strong enough to be stable under denaturing conditions. Proper biotinylation of PC will result in a complete shift on the gel. After confirming the biotinylation, PC was dialyzed against dialysis buffer to remove excess biotin. To yield pure biotinylated PC protein, the protein was purified via a StrepTactin column (IBA, Göttingen, Germany). Therefore, the column was washed with dialysis buffer until the wash fraction appeared clear and PC was eluted with 7 mM biotin. The eluates were pooled and dialyzed one last time against dialysis buffer. The protein was concentrated in a Vivaspin turbo 15 (Sartorius) centrifugal filter device (cutoff, 50 kDa) and stored at −80°C until further use.

### Initial pulldown for identification of potential binding partners.

To identify potential binding partners of PC, B. subtilis was cultivated in 250 ml MSSM medium containing glutamate as the sole nitrogen source and 5 mM potassium chloride until exponential growth phase was reached (OD_600_, ∼0.4 to 0.6). The cells were harvested immediately and stored at −20°C. Naturally biotinylated proteins and their potential interaction partners were then purified from crude extracts using a StrepTactin column (IBA, Göttingen, Germany) and desthiobiotin (2.5 mM) as the eluent. The eluted proteins were analyzed by mass spectrometry analysis.

### *In vitro* analysis of protein-protein interactions.

To study the interaction between DarB and PC, E. coli Rosetta(DE3) was transformed with pGP2972(6×His-SUMO-DarB), pGP3481(6×His-SUMO-DarB^L38F^), pGP3618(6×His-SUMO-YlxR), pGP3458(PC-6×His), or pGP3455(PC^CT+BCCP^-6×His), and the proteins were overexpressed as described above; 250 μl of StrepTactin (IBA, Göttingen, Germany) matrix was equilibrated and loaded with 2 nmol PC and 15 nmol DarB (if stated preincubated 30 min with c-di-AMP [150 nmol]) or 15 nmol YlxR, respectively. Incubation happened overnight at 4°C under constant rotation. Purification was continued by extensive washing of the column with dialysis buffer before PC, together with binding partners, was eluted with biotin (7 mM). The elution fractions were analyzed by Western blotting (50) using antibodies against the His tag (1:1,000) or DarB (1:10,000), respectively, silver staining, and mass spectrometry.

### Protein identification by mass spectrometry and data analysis.

The elution fractions of the pulldown experiments were analyzed by mass spectrometry. For this purpose, the respective samples were separated on a polyacrylamide gel, the respective bands were excised, and the peptides were digested and extracted from the gel pieces and purified using C_18_ stop and go extraction (STAGE) tips as described previously (12, 51–53). The peptides were separated by reverse-phase liquid chromatography using an RSLCnano Ultimate 3000 system (Thermo Fisher Scientific). Peptides were loaded on an Acclaim PepMap 100 precolumn (100 μm by 2 cm, C_18_, 3 μm, 100 Å; Thermo Fisher Scientific) with 0.07% trifluoroacetic acid. Analytical separation of peptides was done using an Acclaim PepMap RSLC column (75 μm by 50 cm, C_18_, 3 μm, 100 Å; Thermo Fisher Scientific) running a water-acetonitrile gradient at a flow rate of 300 nl/min. Chromatographically eluting peptides were online ionized by nano-electrospray ionization (nESI) with a Nanospray flex ion source (Thermo Scientific) and continuously transferred into the mass spectrometer Q Exactive HF (Thermo Scientific). Full scans in a mass range of 300 to 1,650 *m/z* were recorded at a resolution of 30,000, followed by data-dependent top 10 HCD fragmentation at a resolution of 15,000 (dynamic exclusion enabled). Liquid chromatography-mass spectrometry (LC-MS) method programming and data acquisition were performed with XCalibur software 4.0 (Thermo Fisher Scientific). MS/MS2 data were searched against a B. subtilis-specific protein database (UniProt Proteome ID UP000001570) using the Proteome Discoverer Software 2.2. The digestion mode was trypsin/P, and the maximum number of missed cleavage sites was set to two. Carbamidomethyl at cysteines was set as a fixed modification, and oxidation at methionines and N-terminal acetylation of proteins were variable modifications. Mass tolerances of precursors and fragment ions were 10 ppm and 20 ppm, respectively. False discovery rates were calculated using the reverse decoy mode, and the filter for peptide spectrum matches was set to 0.01.

### Isothermal titration calorimetry.

ITC experiments were carried out with a VP-ITC microcalorimeter (MicroCal Inc., Northampton, MA) to determine the affinity of DarB to c-di-AMP or Rel^NTD^. In a typical setup, DarB (10 μM) was placed in the sample cell, and c-di-AMP (100 μM in the same buffer) was placed in the titration syringe. All experiments were carried out at 20°C with a stirring speed of 329 rpm. The parameters used for the titration series are given in Table S4. Data analysis was carried out using MicroCal PEQ-ITC analysis software (MalvernPanalytical).

10.1128/mbio.03602-21.10 TABLE S4 Parameters used for the ITC titration series. Download Table S4, PDF file, 0.04 MB. Copyright © 2022 Krüger et al. 2022 Krüger et al. https://creativecommons.org/licenses/by/4.0/ This content is distributed under the terms of the Creative Commons Attribution 4.0 International license.

### Kinetic studies.

The catalytic activity of PC was assessed by coupling the production of oxaloacetate to the oxidation of NADH by malate dehydrogenase, which can be monitored spectrophotometrically by the decrease in absorbance at 340 nm. The activity was measured at 25°C in a reaction mixture containing 0.26 μM PC, 2.6 μM DarB, DarB^L38F^ or BSA (based on monomer), 10 mM Tris (pH 7.8), 150 mM KCl, 5 mM MgCl_2_, 10 U malate dehydrogenase, 0.4 mM NADH, 25 mM KHCO_3_, 2.5 mM pyruvate, 2 mM ATP, and 100 μM acetyl-CoA, if not stated otherwise. The reaction was started with addition of the substrates.

### Determination of intracellular amino acid and organic acid pools.

Intracellular metabolite levels of B. subtilis cells were determined by GC-MS. Bacterial cells were cultivated in LB medium until exponential growth phase; 40 ml of each culture was harvested by filtration (54). The filter was washed three times with 0.6% NaCl and transferred into 4 ml extraction solution (60% ethanol) and stored overnight at −20°C. *Allo*-inositol was used as the internal standard. Metabolites were extracted as described previously (55). Samples were dried under nitrogen stream and then extracted with 1 ml of extraction buffer (methanol-chloroform-water, 32.25:12.5:6.25 [vol/vol/vol]). To induce phase separation, 500 μl water was added and samples were taken for derivatization from the upper polar fraction. For metabolites of low abundance, 200 μl of the upper phase was evaporated under nitrogen stream while 10 μl was used for metabolites of high abundance. The evaporated samples were derivatized with 15 μl methoxyamine hydrochloride and 30 μl N-methyl-N-(trimethylsilyl) trifluoroacetamide (MSTFA) as described previously (56) to transform the metabolites into their methoxyimino (MEOX) and trimethylsilyl (TMS) derivatives. Samples of low-abundance metabolites were measured with a split of 1:5, and high-abundance metabolite samples were measured with a split of 1:2. GC-MS measurements were performed as described previously (57). If the metabolites were not identified by an external standard, the spectra were identified with the Golm metabolome database (GMD) and the National Institute of Standards and Technology (NIST) spectral library 2.0f. The chemical information on metabolites identified with the GMD can be obtained at http://gmd.mpimp-golm.mpg.de/search.aspx (58). Data were analyzed using MSD ChemStation (F.01.03.2357). The data were analyzed by relative quantification. The average of the wild type was set to 1, and all data were normalized to that. Values between 0 and 1 indicate lower relative abundance and values larger than 1 higher abundance compared to wild-type levels.

### Identification of c-di-AMP-binding proteins by DRaCALA.

The expression of the proteins upon induction with IPTG (final concentration, 0.1 mM) was verified by SDS-PAGE. ^32^P-labeled c-di-AMP synthesis was performed using purified diadenylate cyclase DisA (18). The protein-ligand interaction experiment was performed using E. coli whole-cell lysates, which were either induced by the addition of IPTG (final concentration, 1 mM) overnight or in 1:50 subculture as described previously (59, 60). All binding reactions were performed in 1× binding buffer (10 mM Tris, pH 8, 100 mM NaCl, 5 mM MgCl_2_) containing ∼10 pM ^32^P-labeled c-di-AMP. Protein-ligand mixtures were spotted on nitrocellulose membrane (Amersham Hybond-ECL; GE Healthcare) and dried. The areas and intensities of the spots were quantified by exposing phosphorimager screens and scanning by a FUJI FLA-7000 phosphorimager. The competition assays were performed with 100 μM unlabeled ATP and the absence or presence of 100 μM unlabeled c-di-AMP (Axxora).
