# Supplementary material for: Sustained Control of Pyruvate Carboxylase by the Essential Second Messenger Cyclic di-AMP in Bacillus subtilis
Source: mBio. 2022 Feb 8;13(1):e03602-21. doi: 10.1128/mbio.03602-21 (PMC8822347; doi:10.1128/mbio.03602-21)
Supplement: DATA SET S1 [file mbio.03602-21-sd001.pdf]

Supplementary Data S1

A. **Analysis of eluates from the initial pulldown experiment with *Bacillus subtilis* cell extract**

Peptides identified by search of MS/MS2 data against *B. subtilis* specific protein database (UniProt Proteome ID UP000001570)

**LK59: Wash fraction**				
**Accession**	**Description**	**Coverage (%)**	**Number of identified peptides**	**Number of peptide sequence matches**
P13800	Transcriptional regulatory protein DegU OS=Bacillus subtilis (strain 168) GN=degU PE=1 SV=2	41	7	32
P33166	Elongation factor Tu OS=Bacillus subtilis (strain 168) GN=tuf PE=3 SV=1	38	10	27
P12425	Glutamine synthetase OS=Bacillus subtilis (strain 168) GN=glnA PE=1 SV=3	21	6	19
P28598	60 kDa chaperonin OS=Bacillus subtilis (strain 168) GN=groL PE=1 SV=3	20	8	19
Q9KWU4	Pyruvate carboxylase OS=Bacillus subtilis (strain 168) GN=pyc PE=3 SV=1	11	10	20
P37809	ATP synthase subunit beta OS=Bacillus subtilis (strain 168) GN=atpD PE=1 SV=1	13	5	11
P80868	Elongation factor G OS=Bacillus subtilis (strain 168) GN=fusA PE=1 SV=3	18	9	16
P37808	ATP synthase subunit alpha OS=Bacillus subtilis (strain 168) GN=atpA PE=1 SV=3	13	5	12
P08821	DNA-binding protein HU 1 OS=Bacillus subtilis (strain 168) GN=hupA PE=1 SV=2	32	2	6
P17820	Chaperone protein DnaK OS=Bacillus subtilis (strain 168) GN=dnaK PE=1 SV=3	10	4	10
P80886	Succinate--CoA ligase [ADP-forming] subunit beta OS=Bacillus subtilis (strain 168) GN=sucC PE=1 SV=2	15	5	10
P39583	GTP pyrophosphokinase YwaC OS=Bacillus subtilis (strain 168) GN=ywaC PE=1 SV=1	24	5	16
P02394	50S ribosomal protein L7/L12 OS=Bacillus subtilis (strain 168) GN=rplL PE=1 SV=4	19	2	5
O34347	Argininosuccinate synthase OS=Bacillus subtilis (strain 168) GN=argG PE=3 SV=1	5	2	6
P20277	50S ribosomal protein L17 OS=Bacillus subtilis (strain 168) GN=rplQ PE=1 SV=2	9	1	4
O34633	Uncharacterized protein YjlC OS=Bacillus subtilis (strain 168) GN=yjlC PE=2 SV=1	18	2	6
P09124	Glyceraldehyde-3-phosphate dehydrogenase 1 OS=Bacillus subtilis (strain 168) GN=gapA PE=1 SV=2	7	2	3
P23129	2-oxoglutarate dehydrogenase E1 component OS=Bacillus subtilis (strain 168) GN=odhA PE=3 SV=3	4	3	6

O05252	Uncharacterized lipoprotein YufN OS=Bacillus subtilis (strain 168) GN=yufN PE=3 SV=2	3	1	4
P70974	50S ribosomal protein L13 OS=Bacillus subtilis (strain 168) GN=rplM PE=1 SV=2	13	2	5
P46898	50S ribosomal protein L6 OS=Bacillus subtilis (strain 168) GN=rplF PE=1 SV=2	12	2	5
P37253	Ketol-acid reductoisomerase (NADP(+)) OS=Bacillus subtilis (strain 168) GN=ilvC PE=1 SV=1	8	2	4
P80698	Trigger factor OS=Bacillus subtilis (strain 168) GN=tig PE=1 SV=3	11	3	4
P16263	Dihydrolipoyllysine-residue succinyltransferase component of 2-oxoglutarate dehydrogenase complex OS=Bacillus subtilis (strain 168) GN=odhB PE=3 SV=2	16	6	13
P80861	NADH dehydrogenase-like protein YjlD OS=Bacillus subtilis (strain 168) GN=yjlD PE=1 SV=3	11	3	6
P24327	Foldase protein PrsA OS=Bacillus subtilis (strain 168) GN=prsA PE=1 SV=1	5	1	2
P39912	Protein AroA(G) OS=Bacillus subtilis (strain 168) GN=aroA PE=1 SV=1	18	5	8
P71014	Uncharacterized protein YuaB OS=Bacillus subtilis (strain 168) GN=yuaB PE=1 SV=2	8	1	2
P80877	5-methyltetrahydropteroyltriglutamate--homocysteine methyltransferase OS=Bacillus subtilis (strain 168) GN=metE PE=1 SV=4	9	3	5
O07021	Lactate utilization protein B OS=Bacillus subtilis (strain 168) GN=lutB PE=1 SV=2	3	1	2
P80239	Alkyl hydroperoxide reductase subunit C OS=Bacillus subtilis (strain 168) GN=ahpC PE=1 SV=2	7	1	2
P39148	Serine hydroxymethyltransferase OS=Bacillus subtilis (strain 168) GN=glyA PE=1 SV=1	10	3	5
P37487	Manganese-dependent inorganic pyrophosphatase OS=Bacillus subtilis (strain 168) GN=ppaC PE=1 SV=1	7	2	3
P37527	Pyridoxal 5'-phosphate synthase subunit PdxS OS=Bacillus subtilis (strain 168) GN=pdxS PE=1 SV=3	4	1	2
P39126	Isocitrate dehydrogenase [NADP] OS=Bacillus subtilis (strain 168) GN=icd PE=1 SV=1	9	3	6
P21880	Dihydrolipoyl dehydrogenase OS=Bacillus subtilis (strain 168) GN=pdhD PE=3 SV=1	22	6	11
P04990	Threonine synthase OS=Bacillus subtilis (strain 168) GN=thrC PE=3 SV=1	4	1	2
P38494	30S ribosomal protein S1 homolog OS=Bacillus subtilis (strain 168) GN=ypfD PE=1 SV=1	3	1	2
P21882	Pyruvate dehydrogenase E1 component subunit beta OS=Bacillus subtilis (strain 168) GN=pdhB PE=3 SV=2	6	2	3
O31618	Thiazole synthase OS=Bacillus subtilis (strain 168) GN=thiG PE=1 SV=1	5	1	2
Q06797	50S ribosomal protein L1 OS=Bacillus subtilis (strain 168) GN=rplA PE=1 SV=4	5	1	2
P81100	Stress response protein SCP2 OS=Bacillus subtilis (strain 168) GN=yceC PE=1 SV=3	5	1	2
Q05852	UTP--glucose-1-phosphate uridylyltransferase OS=Bacillus subtilis (strain 168) GN=gtaB PE=1 SV=1	4	1	2

O32157	Fructosamine deglycase FrlB OS=Bacillus subtilis (strain 168) GN=frlB PE=1 SV=1	3	1	2
P53001	Aspartate aminotransferase OS=Bacillus subtilis (strain 168) GN=aspB PE=3 SV=1	7	2	3
P21881	Pyruvate dehydrogenase E1 component subunit alpha OS=Bacillus subtilis (strain 168) GN=pdhA PE=1 SV=3	5	2	3
P49814	Malate dehydrogenase OS=Bacillus subtilis (strain 168) GN=mdh PE=1 SV=3	4	1	2
P12875	50S ribosomal protein L14 OS=Bacillus subtilis (strain 168) GN=rplN PE=1 SV=1	11	1	2
P13243	Probable fructose-bisphosphate aldolase OS=Bacillus subtilis (strain 168) GN=fbaA PE=1 SV=2	18	3	4
P27206	Surfactin synthase subunit 1 OS=Bacillus subtilis (strain 168) GN=srfAA PE=1 SV=4	1	3	5
Q04747	Surfactin synthase subunit 2 OS=Bacillus subtilis (strain 168) GN=srfAB PE=1 SV=3	1	3	5
P08838	Phosphoenolpyruvate-protein phosphotransferase OS=Bacillus subtilis (strain 168) GN=ptsI PE=1 SV=3	4	2	3
P20429	DNA-directed RNA polymerase subunit alpha OS=Bacillus subtilis (strain 168) GN=rpoA PE=1 SV=1	9	2	4
P50849	Polyribonucleotide nucleotidyltransferase OS=Bacillus subtilis (strain 168) GN=pnp PE=1 SV=3	2	1	2
P80859	6-phosphogluconate dehydrogenase, NADP(+)-dependent, decarboxylating OS=Bacillus subtilis (strain 168) GN=gndA PE=1 SV=4	7	2	4
P21883	Dihydrolipoyllysine-residue acetyltransferase component of pyruvate dehydrogenase complex OS=Bacillus subtilis (strain 168) GN=pdhC PE=1 SV=2	4	2	3
P80865	Succinate--CoA ligase [ADP-forming] subunit alpha OS=Bacillus subtilis (strain 168) GN=sucD PE=1 SV=3	5	1	2
P02968	Flagellin OS=Bacillus subtilis (strain 168) GN=hag PE=1 SV=2	7	1	2
P21471	30S ribosomal protein S10 OS=Bacillus subtilis (strain 168) GN=rpsJ PE=1 SV=4	22	2	3
C0SP85	Protein YukE OS=Bacillus subtilis (strain 168) GN=yukE PE=1 SV=1	15	1	2
P54535	Arginine-binding extracellular protein ArtP OS=Bacillus subtilis (strain 168) GN=artP PE=1 SV=1	4	1	2
P46899	50S ribosomal protein L18 OS=Bacillus subtilis (strain 168) GN=rplR PE=1 SV=1	8	1	2
P0CI73	Glutamine--fructose-6-phosphate aminotransferase [isomerizing] OS=Bacillus subtilis (strain 168) GN=glmS PE=2 SV=1	2	1	2
Q04797	Aspartate-semialdehyde dehydrogenase OS=Bacillus subtilis (strain 168) GN=asd PE=1 SV=1	3	1	2
P21465	30S ribosomal protein S3 OS=Bacillus subtilis (strain 168) GN=rpsC PE=1 SV=4	14	2	3
P46349	GABA permease OS=Bacillus subtilis (strain 168) GN=gabP PE=1 SV=3	3	1	2
P12877	50S ribosomal protein L5 OS=Bacillus subtilis (strain 168) GN=rplE PE=1 SV=1	12	1	2

C0H421	Uncharacterized protein YozT OS=Bacillus subtilis (strain 168) GN=yozT PE=4 SV=1	39	1	1
O31980	SPBc2 prophage-derived uncharacterized protein YomE OS=Bacillus subtilis (strain 168) GN=yomE PE=4 SV=1	2	1	1
P80885	Pyruvate kinase OS=Bacillus subtilis (strain 168) GN=pyk PE=1 SV=2	3	1	1
P54161	5'-3' exonuclease OS=Bacillus subtilis (strain 168) GN=ypcP PE=1 SV=1	9	1	1
P42420	6-phospho-5-dehydro-2-deoxy-D-gluconate aldolase OS=Bacillus subtilis (strain 168) GN=iolJ PE=1 SV=1	3	1	2
O07020	Lactate utilization protein A OS=Bacillus subtilis (strain 168) GN=lutA PE=1 SV=1	4	1	1
P37967	Para-nitrobenzyl esterase OS=Bacillus subtilis (strain 168) GN=pnbA PE=1 SV=2	7	1	1
O34364	Probable oligo-1,6-glucosidase 2 OS=Bacillus subtilis (strain 168) GN=ycdG PE=2 SV=1	3	1	1
P94417	Aspartokinase 3 OS=Bacillus subtilis (strain 168) GN=yclM PE=1 SV=1	7	1	1
O30509	Aspartyl/glutamyl-tRNA(Asn/Gln) amidotransferase subunit B OS=Bacillus subtilis (strain 168) GN=gatB PE=1 SV=2	2	1	1
P96711	Arabinose metabolism transcriptional repressor OS=Bacillus subtilis (strain 168) GN=araR PE=1 SV=3	5	1	1
P53556	8-amino-7-oxononanoate synthase 2 OS=Bacillus subtilis (strain 168) GN=bioF PE=1 SV=1	5	1	1
O34948	Uncharacterized oxidoreductase YkwC OS=Bacillus subtilis (strain 168) GN=ykwC PE=3 SV=1	4	1	1
O06997	Uncharacterized FAD-linked oxidoreductase YvdP OS=Bacillus subtilis (strain 168) GN=yvdP PE=1 SV=1	7	1	1
P35155	Segregation and condensation protein B OS=Bacillus subtilis (strain 168) GN=scpB PE=1 SV=1	5	1	2
P37580	Iron(3+)-hydroxamate-binding protein FhuD OS=Bacillus subtilis (strain 168) GN=fhuD PE=1 SV=1	3	1	1
O07596	Putative polysaccharide deacetylase YheN OS=Bacillus subtilis (strain 168) GN=yheN PE=3 SV=1	2	1	1
P54576	Methyl-accepting chemotaxis protein McpC OS=Bacillus subtilis (strain 168) GN=mcpC PE=1 SV=2	4	1	1
460259	enolase [Bacillus subtilis subsp. subtilis str. 168]	2	1	1
O31989	Sublancin immunity protein SunI OS=Bacillus subtilis (strain 168) GN=sunI PE=1 SV=1	11	1	1
O32162	FeS cluster assembly protein SufB OS=Bacillus subtilis (strain 168) GN=sufB PE=3 SV=1	2	1	1
16078208	oligopeptide ABC transporter binding lipoprotein [Bacillus subtilis subsp. subtilis str. 168]	2	1	1
P54518	Uncharacterized peptidase YqhT OS=Bacillus subtilis (strain 168) GN=yqhT PE=3 SV=1	2	1	1
300659766	unnamed protein product [Bacillus subtilis subsp. subtilis str. 168]	6	1	1

LK60: Elution fraction				
**Accession**	**Description**	**Coverage (%)**	**Number of identified peptides**	**Number of peptide sequence matches**
Q9KWU4	Pyruvate carboxylase OS=Bacillus subtilis (strain 168) GN=pyc PE=3 SV=1	52	43	155
O31698	CBS domain-containing protein DarB OS=Bacillus subtilis (strain 168) GN=darB PE=1 SV=1	73	7	104
P13800	Transcriptional regulatory protein DegU OS=Bacillus subtilis (strain 168) GN=degU PE=1 SV=2	57	10	23
P39583	GTP pyrophosphokinase YwaC OS=Bacillus subtilis (strain 168) GN=ywaC PE=1 SV=1	24	5	18
P33166	Elongation factor Tu OS=Bacillus subtilis (strain 168) GN=tuf PE=3 SV=1	22	6	14
P39912	Protein AroA(G) OS=Bacillus subtilis (strain 168) GN=aroA PE=1 SV=1	19	5	10
P49786	Biotin carboxyl carrier protein of acetyl-CoA carboxylase OS=Bacillus subtilis (strain 168) GN=accB PE=1 SV=2	35	3	9
P17820	Chaperone protein DnaK OS=Bacillus subtilis (strain 168) GN=dnaK PE=1 SV=3	7	3	8
P37809	ATP synthase subunit beta OS=Bacillus subtilis (strain 168) GN=atpD PE=1 SV=1	12	4	8
P46898	50S ribosomal protein L6 OS=Bacillus subtilis (strain 168) GN=rplF PE=1 SV=2	28	3	5
P16263	Dihydrolipoyllysine-residue succinyltransferase component of 2-oxoglutarate dehydrogenase complex OS=Bacillus subtilis (strain 168) GN=odhB PE=3 SV=2	8	3	5
P28598	60 kDa chaperonin OS=Bacillus subtilis (strain 168) GN=groL PE=1 SV=3	7	2	4
P71014	Uncharacterized protein YuaB OS=Bacillus subtilis (strain 168) GN=yuaB PE=1 SV=2	8	1	4
P27206	Surfactin synthase subunit 1 OS=Bacillus subtilis (strain 168) GN=srfAA PE=1 SV=4	1	3	4
P37808	ATP synthase subunit alpha OS=Bacillus subtilis (strain 168) GN=atpA PE=1 SV=3	7	3	4
P80698	Trigger factor OS=Bacillus subtilis (strain 168) GN=tig PE=1 SV=3	9	2	3

P02394	50S ribosomal protein L7/L12 OS=Bacillus subtilis (strain 168) GN=rplL PE=1 SV=4	19	2	3
P21880	Dihydrolipoyl dehydrogenase OS=Bacillus subtilis (strain 168) GN=pdhD PE=3 SV=1	12	3	3
P80886	Succinate--CoA ligase [ADP-forming] subunit beta OS=Bacillus subtilis (strain 168) GN=sucC PE=1 SV=2	3	1	2
P12877	50S ribosomal protein L5 OS=Bacillus subtilis (strain 168) GN=rplE PE=1 SV=1	12	1	2
P80868	Elongation factor G OS=Bacillus subtilis (strain 168) GN=fusA PE=1 SV=3	2	1	2
P39126	Isocitrate dehydrogenase [NADP] OS=Bacillus subtilis (strain 168) GN=icd PE=1 SV=1	3	1	2
P37571	Negative regulator of genetic competence ClpC/MecB OS=Bacillus subtilis (strain 168) GN=clpC PE=1 SV=1	2	1	2
P70974	50S ribosomal protein L13 OS=Bacillus subtilis (strain 168) GN=rplM PE=1 SV=2	7	1	2
P80244	ATP-dependent Clp protease proteolytic subunit OS=Bacillus subtilis (strain 168) GN=clpP PE=1 SV=3	7	1	2
P39751	MreB-like protein OS=Bacillus subtilis (strain 168) GN=mbl PE=3 SV=1	3	1	2
Q01465	Rod shape-determining protein MreB OS=Bacillus subtilis (strain 168) GN=mreB PE=3 SV=3	3	1	2
Q05852	UTP--glucose-1-phosphate uridylyltransferase OS=Bacillus subtilis (strain 168) GN=gtaB PE=1 SV=1	8	2	2
P12425	Glutamine synthetase OS=Bacillus subtilis (strain 168) GN=glnA PE=1 SV=3	8	2	2
300659766	unnamed protein product [Bacillus subtilis subsp. subtilis str. 168]	6	1	2
P37947	HTH-type transcriptional regulator DegA OS=Bacillus subtilis (strain 168) GN=degA PE=1 SV=1	4	1	1
P81100	Stress response protein SCP2 OS=Bacillus subtilis (strain 168) GN=yceC PE=1 SV=3	5	1	1
O34324	Dihydrolipoyl dehydrogenase OS=Bacillus subtilis (strain 168) GN=acoL PE=3 SV=1	2	1	1
O07596	Putative polysaccharide deacetylase YheN OS=Bacillus subtilis (strain 168) GN=yheN PE=3 SV=1	2	1	1
P28612	Motility protein B OS=Bacillus subtilis (strain 168) GN=motB PE=3 SV=1	11	1	1
P40872	Polyketide synthase PksM OS=Bacillus subtilis (strain 168) GN=pksM PE=1 SV=4	1	1	1

P21464	30S ribosomal protein S2 OS=Bacillus subtilis (strain 168) GN=rpsB PE=1 SV=3	5	1	1
1296452	ClpX protein [Bacillus subtilis subsp. subtilis str. 168]	3	1	1
O32157	Fructosamine deglycase FrlB OS=Bacillus subtilis (strain 168) GN=frlB PE=1 SV=1	3	1	1
P12042	Phosphoribosylformylglycinamidine synthase subunit PurL OS=Bacillus subtilis (strain 168) GN=purL PE=1 SV=2	1	1	1
P23129	2-oxoglutarate dehydrogenase E1 component OS=Bacillus subtilis (strain 168) GN=odhA PE=3 SV=3	1	1	1
853769	transcriptional terminator Rho [Bacillus subtilis subsp. subtilis str. 168]	2	1	1
P21471	30S ribosomal protein S10 OS=Bacillus subtilis (strain 168) GN=rpsJ PE=1 SV=4	15	1	1
P38494	30S ribosomal protein S1 homolog OS=Bacillus subtilis (strain 168) GN=ypfD PE=1 SV=1	3	1	1
P80859	6-phosphogluconate dehydrogenase, NADP(+)-dependent, decarboxylating OS=Bacillus subtilis (strain 168) GN=gndA PE=1 SV=4	2	1	1
P54537	Arginine transport ATP-binding protein ArtM OS=Bacillus subtilis (strain 168) GN=artM PE=1 SV=1	3	1	1

B. **Analysis of eluates from the pulldown experiment with PC**

Peptides identified by search of MS/MS2 data against *B. subtilis* specific protein database (UniProt Proteome ID UP000001570)

**LK72: Band 3 from the elution fraction from the column containing PC and YlxR**				
**Accession**	**Description**	**Coverage (%)**	**Number of identified peptides**	**Number of peptide sequence matches**
Q9KWU4	Pyruvate carboxylase OS=Bacillus subtilis (strain 168) GN=pyc PE=3 SV=1	31	26	72
P32728	Uncharacterized protein YlxR OS=Bacillus subtilis (strain 168) GN=ylxR PE=4 SV=1	57	5	8
O34921	Uncharacterized protein YtoI OS=Bacillus subtilis (strain 168) GN=ytoI PE=3 SV=1	2	1	1
O34577	Probable adenylyl-sulfate kinase OS=Bacillus subtilis (strain 168) GN=cysC PE=2 SV=1	4	1	1
P54518	Uncharacterized peptidase YqhT OS=Bacillus subtilis (strain 168) GN=yqhT PE=3 SV=1	2	1	1
P80872	General stress protein 16O OS=Bacillus subtilis (strain 168) GN=yocK PE=1 SV=3	5	1	1
P37580	Iron(3+)-hydroxamate-binding protein FhuD OS=Bacillus subtilis (strain 168) GN=fhuD PE=1 SV=1	3	1	1

**LK73: Band 1 from the elution fraction from the column containing PC and DarB**				
**Accession**	**Description**	**Coverage (%)**	**Number of identified peptides**	**Number of peptide sequence matches**
Q9KWU4	Pyruvate carboxylase OS=Bacillus subtilis (strain 168) GN=pyc PE=3 SV=1	48	45	194
O31698	CBS domain-containing protein DarB OS=Bacillus subtilis (strain 168) GN=darB PE=1 SV=1	68	6	54
Q45066	DNA topoisomerase 4 subunit A OS=Bacillus subtilis (strain 168) GN=parC PE=3 SV=2	2	1	22

LK74: Band 2 from the elution fraction from the column containing PC, DarB and c-di-AMP				
**Accession**	**Description**	**Coverage (%)**	**Number of identified peptides**	**Number of peptide sequence matches**
Q9KWU4	Pyruvate carboxylase OS=Bacillus subtilis (strain 168) GN=pyc PE=3 SV=1	48	44	275
O31698	CBS domain-containing protein DarB OS=Bacillus subtilis (strain 168) GN=darB PE=1 SV=1	49	4	18
Q45066	DNA topoisomerase 4 subunit A OS=Bacillus subtilis (strain 168) GN=parC PE=3 SV=2	2	1	12

C. Analysis of eluates from the pulldown experiment with $PC^{CT+BCCP}$

Peptides identified by search of MS/MS2 data against *B. subtilis* specific protein database (UniProt Proteome ID UP000001570)

**LK75: Band 4 from the elution fraction from the column containing $PC^{CT+BCCP}$ and DarB**				
**Accession**	**Description**	**Coverage (%)**	**Number of identified peptides**	**Number of peptide sequence matches**
Q9KWU4	Pyruvate carboxylase OS=Bacillus subtilis (strain 168) GN=pyc PE=3 SV=1	38	32	346
O31698	CBS domain-containing protein DarB OS=Bacillus subtilis (strain 168) GN=darB PE=1 SV=1	86	7	39
Q45066	DNA topoisomerase 4 subunit A OS=Bacillus subtilis (strain 168) GN=parC PE=3 SV=2	2	1	15

**LK76: Band 5 from the elution fraction from the column containing $PC^{CT+BCCP}$ and YlxR**				
**Accession**	**Description**	**Coverage (%)**	**Number of identified peptides**	**Number of peptide sequence matches**
Q9KWU4	Pyruvate carboxylase OS=Bacillus subtilis (strain 168) GN=pyc PE=3 SV=1	45	39	171
P32728	Uncharacterized protein YlxR OS=Bacillus subtilis (strain 168) GN=ylxR PE=4 SV=1	70	8	17
O34863	UvrABC system protein A OS=Bacillus subtilis (strain 168) GN=uvrA PE=3 SV=1	2	1	1
O32037	tRNA threonylcarbamoyladenosine dehydratase OS=Bacillus subtilis (strain 168) GN=tcdA PE=3 SV=3	2	1	1
P21879	Inosine-5'-monophosphate dehydrogenase OS=Bacillus subtilis (strain 168) GN=guaB PE=1 SV=2	1	1	1
P42954	Teichoic acids export ATP-binding protein TagH OS=Bacillus subtilis (strain 168) GN=tagH PE=1 SV=1	7	1	1
O31902	Probable DNA polymerase YorL OS=Bacillus subtilis (strain 168) GN=yorL PE=3 SV=1	3	1	1
