# Supplementary material for: Sustained Control of Pyruvate Carboxylase by the Essential Second Messenger Cyclic di-AMP in Bacillus subtilis
Source: mBio. 2022 Feb 8;13(1):e03602-21. doi: 10.1128/mbio.03602-21 (PMC8822347; doi:10.1128/mbio.03602-21)
Supplement: FIG S1 [file mbio.03602-21-sf001.pdf]

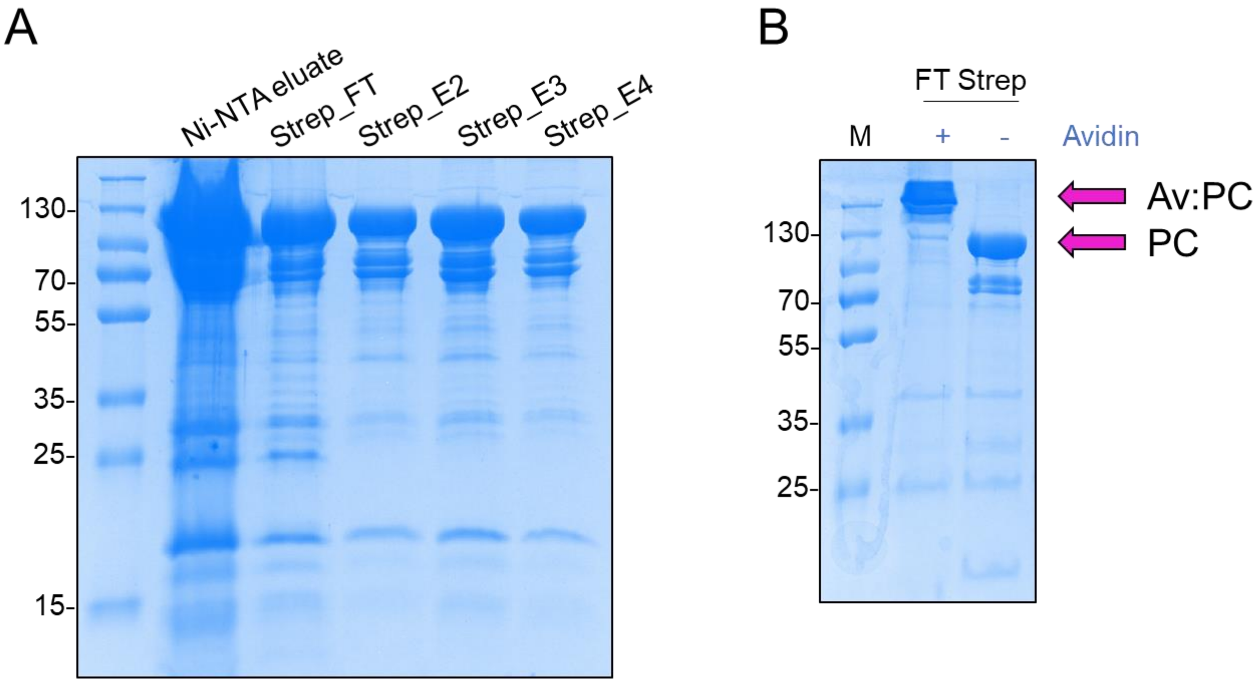

Fig. S1 Purification of PC. (A) PC was purified in a two-step purification protocol first via a NiNTA-column, followed by purification of naturally biotinylated PC via a StrepTactin column. Unbiotinlyated PC was biotinylated *in vitro* by incubation with purified BirA protein. (B) The successful biotinylation of the previously unbiotinylated PC protein from the FT of the StrepTactin purification was tested with an avidin-shift assay.
