# Supplementary material for: Sustained Control of Pyruvate Carboxylase by the Essential Second Messenger Cyclic di-AMP in Bacillus subtilis
Source: mBio. 2022 Feb 8;13(1):e03602-21. doi: 10.1128/mbio.03602-21 (PMC8822347; doi:10.1128/mbio.03602-21)
Supplement: FIG S2 [file mbio.03602-21-sf002.pdf]

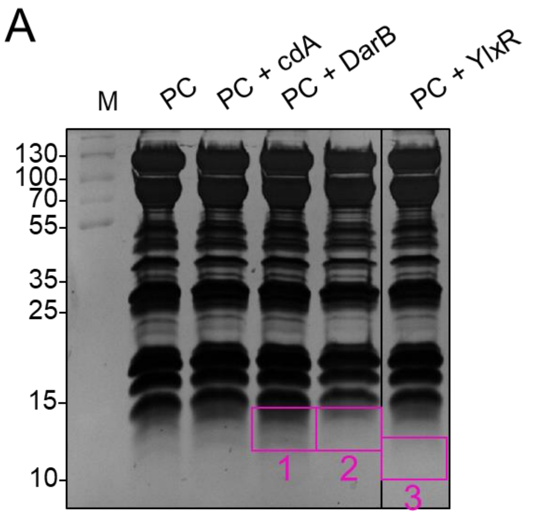

	Description	#PSMs	#Peptides
1	DarB	54	6
2	DarB^{cdA}	18	4
3	YlxR	8	5

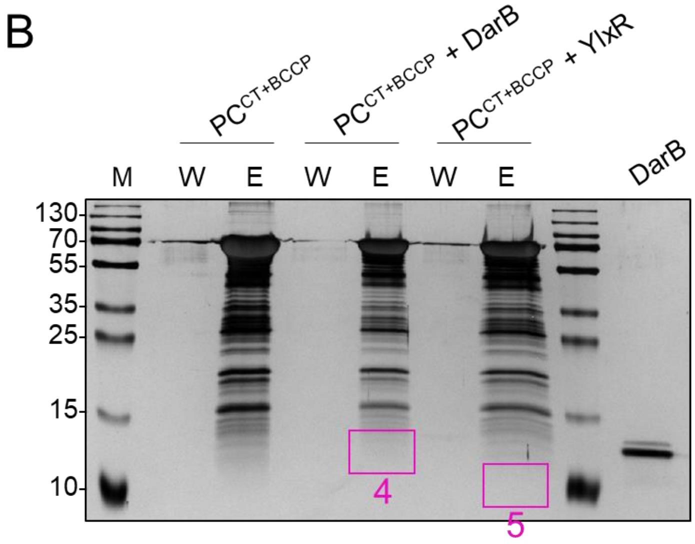

	Description	#PSMs	#Peptides
4	DarB	39	7
5	YlxR	17	8

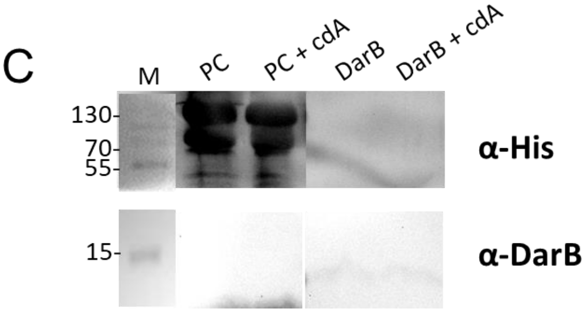

Fig. S2 DarB interacts with PC. SDS-PAGE from *in vitro* pulldown experiments with PC (A) and PC$^{CT+BCCP}$ (B). Purified PC protein was immobilized onto a StrepTactin column and incubated with DarB, DarB preincubated with c-di-AMP, or the control protein YlxR. The presence of DarB and PC in the elution fractions was analysed by SDS-PAGE and the bands were excised from the gel (magenta boxes) and analyzed by mass spectrometry. The amount of DarB or YlxR identified in the excised gel bands is shown in the table. (C). Purified biotinylated His-PC and DarB were passed over a StrepTactin column, and the elution fractions were tested for the presence of His-PC (upper panel) and DarB (lower panel). Abbreviations: PSMs, peptide sequence matches; cdA, c-di-AMP.
