# Supplementary material for: Sustained Control of Pyruvate Carboxylase by the Essential Second Messenger Cyclic di-AMP in Bacillus subtilis
Source: mBio. 2022 Feb 8;13(1):e03602-21. doi: 10.1128/mbio.03602-21 (PMC8822347; doi:10.1128/mbio.03602-21)
Supplement: FIG S3 [file mbio.03602-21-sf003.pdf]

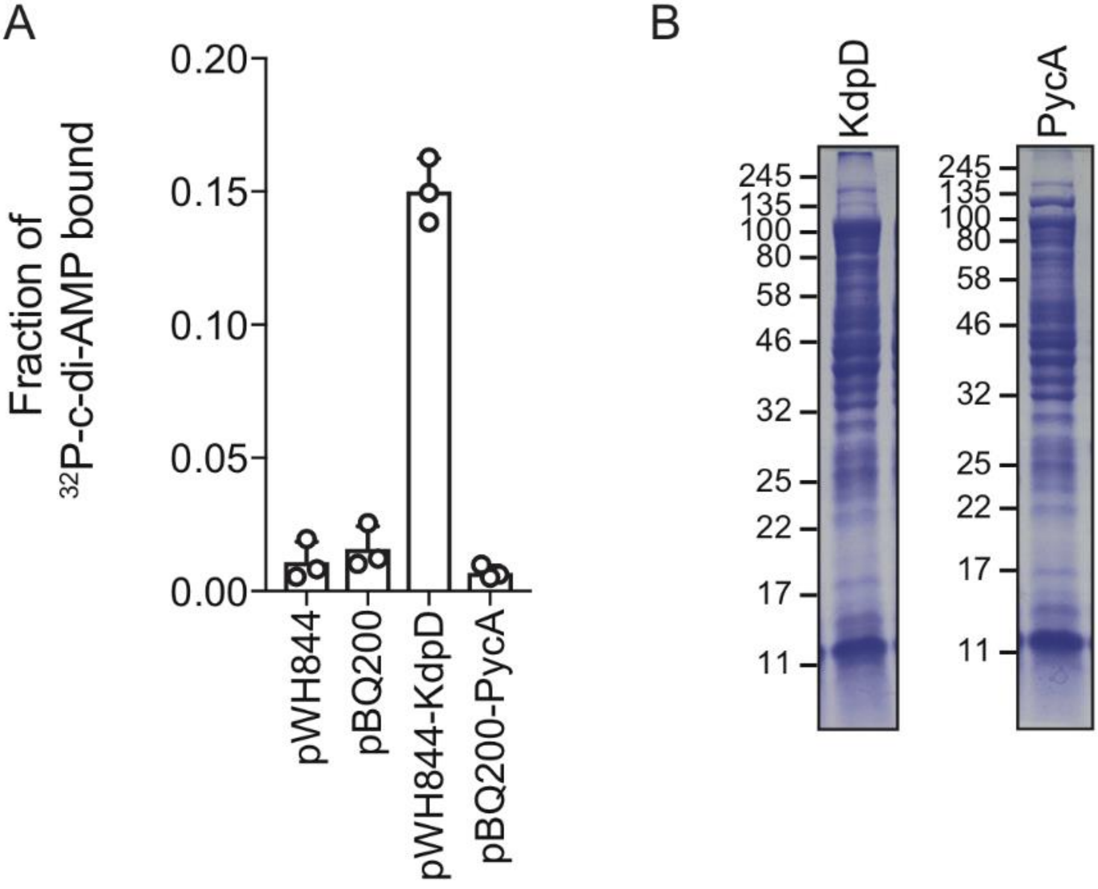

Fig. S3 *E. coli* whole cell lysates overexpressing KdpD but not PycA bind c-di-AMP. (A) Fraction bound of radiolabeled ^{32}P-c-di-AMP from DRaCALA experiments is shown for lysates from *E. coli* induced for the expression of the indicated genes in the presence of 100 µM non-specific ATP competitor. Three independent lysates were prepared and analyzed for each indicated gene and vector controls. (B) Protein expression in *E. coli* lysates induced for expression of the indicated genes used in DRaCALA. Proteins were analyzed by SDS-PAGE and stained by Coomassie Brilliant Blue.
