# Supplementary material for: Sustained Control of Pyruvate Carboxylase by the Essential Second Messenger Cyclic di-AMP in Bacillus subtilis
Source: mBio. 2022 Feb 8;13(1):e03602-21. doi: 10.1128/mbio.03602-21 (PMC8822347; doi:10.1128/mbio.03602-21)
Supplement: FIG S4 [file mbio.03602-21-sf004.pdf]

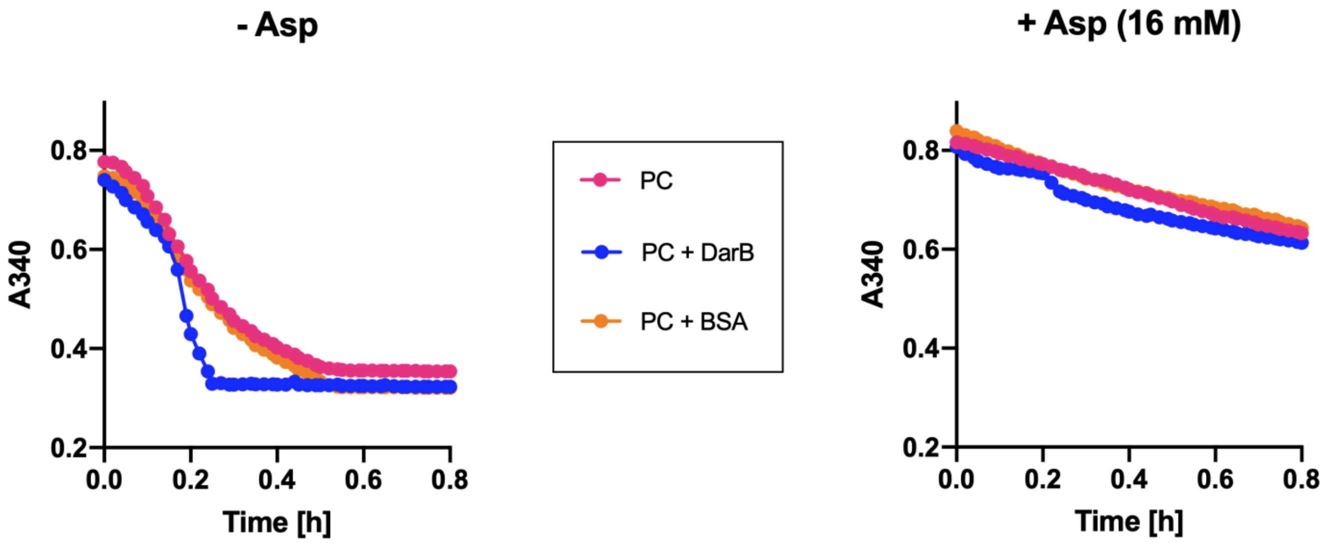

Fig. S4 Biochemical characterization of BsPC regulation by aspartate. The reaction contained 0.26 µM BsPC, 2.6 µM DarB, or BSA (based on monomer), 10 mM Tris (pH 7.8), 150 mM KCl, 5 mM $MgCl_2$, 10 units of malate dehydrogenase, 0.4 mM NADH, 25 mM $KHCO_3$, 2.5 mM pyruvate and 100 µM acetyl-CoA and 16 mM aspartate if indicated. The graph shows the consumption of NADH over time, which is proportional to the synthesis of oxaloacetate by PC.
