# Supplementary material for: Sustained Control of Pyruvate Carboxylase by the Essential Second Messenger Cyclic di-AMP in Bacillus subtilis
Source: mBio. 2022 Feb 8;13(1):e03602-21. doi: 10.1128/mbio.03602-21 (PMC8822347; doi:10.1128/mbio.03602-21)
Supplement: FIG S5 [file mbio.03602-21-sf005.pdf]

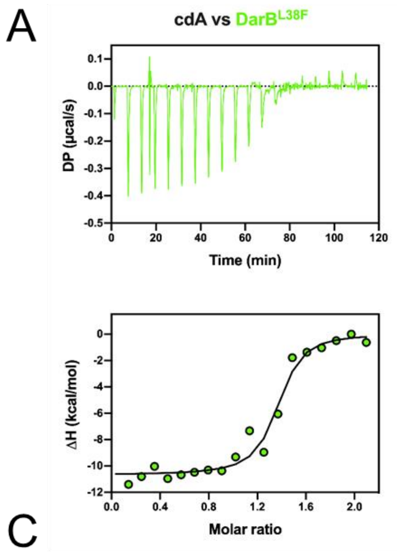

	K_D [M] cdA	N (sites)
DarB	$45.5 \times 10^{-9} \pm 5.67 \times 10^{-9}$	$1.26 \pm 5.3 \times 10^{-3}$
DarB^{L38F}	$72.6 \times 10^{-9} \pm 37.2 \times 10^{-9}$	$1.33 \pm 2.8 \times 10^{-2}$

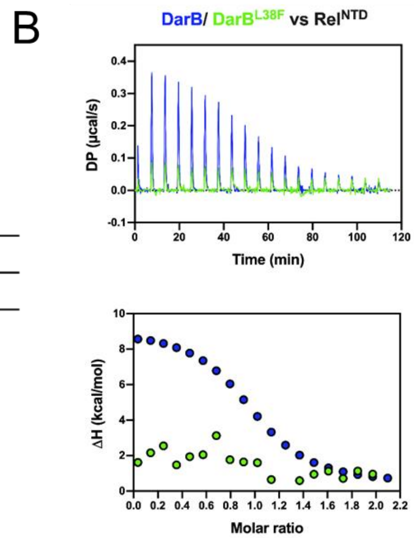

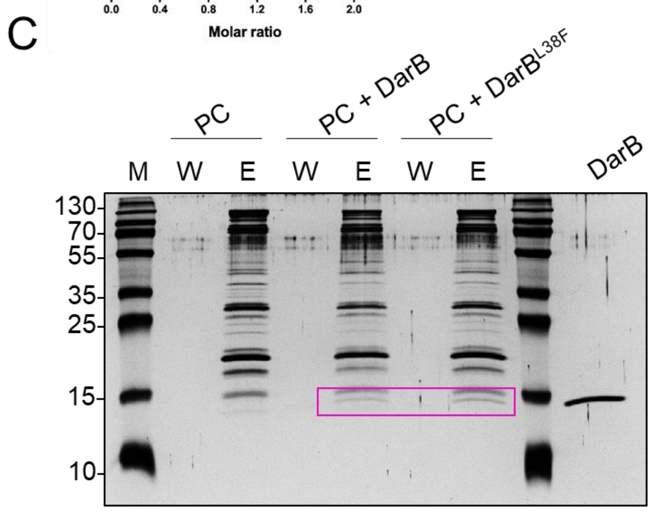

Fig. S5 Analysis of the binding affinities of the DarB mutant L38F towards c-di-AMP and Rel^{NTD}. (A) The ability of the DarB^{L38F} mutant protein to bind c-di-AMP was assessed by Isothermal titration calorimetry (ITC). The cell and the syringe contained 10 µM DarB^{L38F} and 100 µM c-di-AMP, respectively. Titration profiles and the determined molar ratio are shown together with the calculated K_D value for binding of c-di-AMP, as well as the determined number of binding ligand sites. For comparison, the calculated K_D value and number of binding ligand sites from wild type DarB from Krüger 2021 are shown in the table as well. (B) The ability of the DarB^{L38F} mutant (green) to bind Rel was assessed by ITC. The cell and the syringe contained 10 µM Rel^{NTD} and 100 µM DarB^{L38F}, respectively. For comparison, the titration profile of wild type DarB into Rel were taken from Krüger et al 2021 (blue). Titration profiles and the determined molar ratio are shown. (C) Silver stain of SDS-PAGE from *in vitro* pulldown experiments with PC. Purified PC protein was immobilized onto a StrepTactin column and incubated with DarB or the DarB^{L38F} mutant. The elution fractions were analyzed by SDS-PAGE. The magenta box shows the band corresponding to the DarB protein. Abbreviation: cdA, c-di-AMP.
