# Supplementary material for: Sustained Control of Pyruvate Carboxylase by the Essential Second Messenger Cyclic di-AMP in Bacillus subtilis
Source: mBio. 2022 Feb 8;13(1):e03602-21. doi: 10.1128/mbio.03602-21 (PMC8822347; doi:10.1128/mbio.03602-21)
Supplement: TABLE S1 [file mbio.03602-21-st001.pdf]

Supplementary Table S1

Strains used in this study

Name	Genotype	Construction	Reference
168	*trpC2*		
GP797	*trpC2 ΔcitZ::spec*	LFH-PCR → 168	This study
GP2222	*trpC2 ΔcdaA::cat ΔcdaS::ermC ΔdisA::tet*	GP997 → GP991	(1)
GP2769	*trpC2 ΔdarB::ermC*	BKE14130 → 168	(2)
GP2777	*trpC2 ΔcdaS::spc ΔdisA::tet ΔykuL::ermC*	BKE14130 → GP1361	This study
GP2779	*trpC2 ΔcdaS::spc ΔdisA::tet ΔykuL::ermC ΔcdaA::cat*	GP997 → GP2777	This study
GP3706	*trpC2 ΔdarB::cat ΔcitZ::spec*	GP797 → GP3409	This study

1. Gundlach J., Herzberg C., Kaever V., Gunka K., Hoffmann T., Weiß M., Gibhardt J., Thürmer A., Hertel D., Daniel R. *et al.* Control of potassium homeostasis is an essential function of the second messenger cyclic di-AMP in *Bacillus subtilis*. *Sci. Signal*. 2017; 10: eaal3011.
2. Krüger L., Herzberg C., Wicke D., Bähre H., Heidemann J.L., Dickmanns A., Schmitt K., Ficner A., Stülke J. A meet-up of two second messengers: the c-di-AMP receptor DarB controls (p)ppGpp synthesis in *Bacillus subtilis*. *Nat. Commun*. 2021; 12: 1210.
