# Supplementary material for: Sustained Control of Pyruvate Carboxylase by the Essential Second Messenger Cyclic di-AMP in Bacillus subtilis
Source: mBio. 2022 Feb 8;13(1):e03602-21. doi: 10.1128/mbio.03602-21 (PMC8822347; doi:10.1128/mbio.03602-21)
Supplement: TABLE S3 [file mbio.03602-21-st003.pdf]

Supplementary Table S3

Oligonucleotides used in this study

Name	Sequence	Gene	Comments
CZ114	CAGAATTAAAACAAGCATGGC	fwd upstream primer; LFH-PCR for deletion of *citZ*	
CZ115	CCTATCACCTCAAATGGTTCGCTGGTATCATCAATAATAGAACTAAC	rev upstream primer; LFH-PCR for deletion of *citZ*	
CZ116	CATGTCCTAGCTTATCAGAAC	rev downstream primer; LFH-PCR for deletion of *citZ*	
CZ117	CATCGTGATGAAAACGAGGA	Sequencing primer deletion of *citZ*	
CZ118	CAATGTTTTCAATGACTGTCAG	Sequencing primer deletion of *citZ*	
CZ119	GAAAACAATATGCAACTTTAAATC	Sequencing primer deletion of *citZ*	
CZ132	CGAGCGCCTACGAGGAATTTGTATCGCAGATTACACAGGCCCTGAC	fwd downstream primer; LFH-PCR for deletion of *citZ*	
DW40	AAAGGTCTC**ATGGT**GTGAATAAACACAAAAAGATCCCATTACG	fwd, *ylxR* in pET-SUMO	BsaI; **ATGGT** extra for proper cloning
DW41	TTTCTCGAGTTATTTTTTCACCTTTTCCGCCAGTT	rev, *ylxR* in pET-SUMO	XhoI
FR124	AAATCTAGATTGTCTCAGCAATCGATACAAAAAGT	fwd, *pycA* in pBQ200	XbaI
FR125	TTTGCATGCTTATGCTTTTTCAATTTCAAGGAGCAG	rev, *pycA* in pBQ200	SphI
JN335	AAAGGATCCATGCGGTCAACATTAAGAAAAGACCTTATTGAATTATTTTCTC	fwd, *birA* in pWH844	BamHI
JN336	TTTGTCGACTTAGCCCAATTCGATATCGGCAGAATAGATTTTTTTAATG	rev, *birA* in pWH844	SalI
LK80	AAAGGTCTC**ATGGT**ATGATAAGCTTACAATCAGATCAACTTCTTGAGG	fw, *darB* for pET-SUMO	BsaI; **ATGGT** extra for proper cloning
LK81	TTTCTCGAGCTACTTATTCAATGAGCGTATATGCTTATTCAATTCC	rev, *darB* for pET-SUMO	XhoI
LK209	AAATCTAGA**ATTAAAGAGGAGAAATTAACT**ATGATAAGCTTACAATCAGATCAACTTCTTGAGG	fw, *darB* in pBQ200	XbaI, + RBS

LK234	AAATCTAGA**ATTAAAGAGGAGAAATTAACT**TTGTCTCAGCAATCGATACAAAAAGTATTA	fw, *pycA,* pET28a	XbaI, + RBS
LK235	TTTCTCGAGTGCTTTTTCAATTTCAAGGAGCAGATC	rev, *pycA,* pET28a	XhoI
LK421	[Phos]CCTTGAGCATGCATTATTTGTATTGACAAAAACCG	CCR-Primer *darB* (L38F)	5´-P
LK457	AAATCTAGA**ATTAAAGAGGAGAAATTAACTATG**CCTAAACAAAAAGACCGCGGAAC	fw, *pycA* (CT+BCCP)*,* pET28a	XbaI, + RBS, + ATG
TK02	TTTCTGCAGCTACTTATTCAATGAGCGTATATGCTTATTCA	rev, *darB* in pBQ200	PstI

Underlined: restriction sites; bold: additional bases/mutation: [Phos]: 5’-phoshorylation; RBS: ribosomal binding site

Abbreviations: RBS, ribosomal binding site.
