# Supplementary material for: Sustained Control of Pyruvate Carboxylase by the Essential Second Messenger Cyclic di-AMP in Bacillus subtilis
Source: mBio. 2022 Feb 8;13(1):e03602-21. doi: 10.1128/mbio.03602-21 (PMC8822347; doi:10.1128/mbio.03602-21)
Supplement: TABLE S2 [file mbio.03602-21-st002.pdf]

Supplementary Table S2

Plasmids used in this study

**Name**	**Vector**	**Insert**	**Reference**
pWH844			(1)
pBQ200			(2)
pBP560	pWH844	*kdpD*	(3)
pGP1289	pBQ380/ *XbaI* + *SphI*	*pycA* FR124 + FR125 (*XbaI* + *SphI*)	This study
pGP2960	pWH844/ *BamH*I + *Sal*I	PCR product *birA* JN335 + JN336 (*BamHI+SalI*)	This study
pGP2972	pET-SUMO	*darB*	(4)
pGP3306	pBQ200	*darB*	(4)
pGP3429	pWH844	rel^{NTD}	(4)
pGP3481	pET-SUMO/ *Bsa*I + *Xho*I	PCR product *darB*-L38F, LK80 + LK81 + LK421 (*BsaI* + *XhoI*)	This study
pGP3455	pET28a/ *XbaI* + *XhoI*	PCR product *pycA* (CT+BCCP), LK457 + LK235 (*XbaI* + *XhoI*)	This study
pGP3458	pET28a/ *XbaI* + *XhoI*	PCR product *pycA,* LK234 + LK235 (*XbaI* + *XhoI*)	This study
pGP3618	pET-SUMO/ *Bsa*I + *Xho*I	PCR product *ylxR* DW40 + DW41 (*Bsa*I + *Xho*I)	This study

1. Schirmer F., Ehrt, S., Hillen, W. Expression, inducer spectrum, domain structure, and function of MopR, the regulator of phenol degradation in Acinetobacter calcoaceticus NCIB8250. *J. Bacteriol.* 1997; 179: 1329-1336.
2. Martin-Verstraete I., Débarbouillé M., Klier A., Rapoport G. Interactions of wild-type and truncated LevR of Bacillus subtilis with the upstream activating sequence of the levanase operon. *J. Mol. Biol.* 1994; 241: 178-192.
3. Gibhardt J., Hoffmann G., Turdiev A., Wang M., Lee V.T., Commichau F.M. c-di-AMP assists osmoadaptation by regulating the Listeria monocytogenes potassium transporters KimA and KtrCD. *J. Biol. Chem*. 2019; 294: 16020-16033.
4. Krüger L., Herzberg C., Wicke D., Bähre H., Heidemann J.L., Dickmanns A., Schmitt K., Ficner A., Stülke J. A meet-up of two second messengers: the c-di-AMP receptor DarB controls (p)ppGpp synthesis in *Bacillus subtilis*. *Nat. Commun*. 2021; 12: 1210.
