# Supplementary material for: Sustained Control of Pyruvate Carboxylase by the Essential Second Messenger Cyclic di-AMP in Bacillus subtilis
Source: mBio. 2022 Feb 8;13(1):e03602-21. doi: 10.1128/mbio.03602-21 (PMC8822347; doi:10.1128/mbio.03602-21)
Supplement: TABLE S4 [file mbio.03602-21-st004.pdf]

Supplementary Table S4

Parameters used for the ITC titration series

**Injection no.**	**Injection volume [µl]**	**Injection duration [sec]**	**Spacing [sec]**	**Filter period [sec]**
1	5	10	360	2
2-19	15	30	360	2
